# Diatoms vs dinoflagellates: a temporal network analysis of bloom impacts on phytoplankton diversity and community structure in French coastal waters

**DOI:** 10.1093/ismeco/ycag174

**Published:** 2026-06-19

**Authors:** Jean-Yves Dias, Victor Pochic, Samuel Chaffron, Pierre Gernez

**Affiliations:** Institut des Substances et Organismes de la Mer, ISOMER, UR 2160, Nantes Université, Nantes F-44000, France; Laboratoire de Biologie des Organismes et des Écosystèmes Aquatiques-BOREA, Muséum national d’Histoire naturelle (MNHN), SU, CNRS, IRD, UA, Paris F-75005, France; Institut des Substances et Organismes de la Mer, ISOMER, UR 2160, Nantes Université, Nantes F-44000, France; COAST, Ifremer, Nantes F-44000, France; École Centrale Nantes, CNRS, LS2N, UMR 6004, Nantes Université, Nantes F-44000, France; FR2022/Tara Oceans GOSEE, Research Federation for the Study of Global Ocean Systems Ecology and Evolution, Paris F-75016, France; Institut des Substances et Organismes de la Mer, ISOMER, UR 2160, Nantes Université, Nantes F-44000, France

**Keywords:** phytoplankton blooms, temporal networks, community structure, diatoms, dinoflagellates, phytoplankton time-series

## Abstract

Understanding phytoplankton community responses to bloom events is essential as these have major implications for biogeochemical cycles and marine food webs, but can also be harmful to ecosystems, the economy, and public health. Diatoms and dinoflagellates are the two most common bloom-forming classes, with distinct ecologies and bloom dynamics. Using time series from a long-term phytoplankton monitoring survey (REPHY) of French coastal waters analyzed with ecological association networks and diversity metrics, we investigated how blooms of diatoms and dinoflagellates affect diversity and community structure within phytoplankton communities. We highlight distinct responses: an increase in species richness during diatom blooms and a decrease during dinoflagellate blooms. However, both bloom types resulted in a decrease in Shannon and Pielou indices. Temporal association network modularity and association strength were also differently impacted between the two bloom types. However, high-level association types and composition in association networks remain stable, suggesting an important role of their proportions in ecosystem functioning and resilience. We propose several hypotheses for these findings based on known ecological and biological processes that remain to be tested individually in dedicated studies. Our work underlines the importance of distinguishing the different types of blooms and studying the role of interactions between phytoplankton for a better understanding of their dynamics.

## Graphical abstract

**Graphical Abstract:**
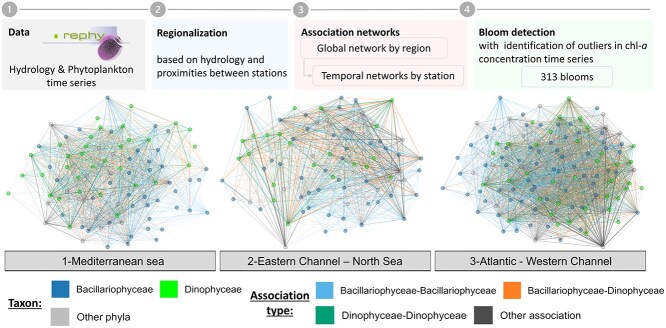

## Introduction

For decades, phytoplankton has been known to play a major role in marine ecosystems as a primary producer [1]. It is therefore crucial to develop an advanced understanding of phytoplankton dynamics and particularly bloom events. Bloom studies remain a complex task due to the lack of consensus on what constitutes a bloom. Most studies define blooms as an exceptional biomass, in line with the definition provided by the International Council for the Exploration of the Sea (ICES), which describes blooms as a deviation from the “normal cycle” of phytoplankton biomass [2, 3]. Other definitions rely on thresholds of chlorophyll-*a* concentration [4] or cell abundance [5], often defined arbitrarily. Some researchers criticize threshold-based approaches and argue that the ICES definition is also inadequate for seasonal blooms, as they are part of the “normal cycle” [6].

Several hypotheses have been proposed to understand bloom formation, most of them relying on abiotic conditions [7–10]. More recently, biotic interactions with zooplankton or bacterioplankton have been considered to explain this phenomenon [11–14]. In temperate ecosystems, phytoplankton blooms are expected to occur during spring and to be dominated by large and fast-growing diatoms. It can be followed by shorter summer blooms mainly dominated by smaller diatoms, dinoflagellates, and other flagellates [15]. Diatoms and dinoflagellates are the most common bloom-forming classes [15]. Diatoms (class Bacillariophyceae) are generally permanent species with a cosmopolitan distribution. Their blooms are relatively predictable and cyclical. In contrast, dinoflagellates are characterized by short, massive occurrences with high interannual variability. Dinoflagellates (class Dinophyceae) display significant ecophysiological diversity, often occupying restricted ecological niches [16]. Some species can dominate the phytoplankton community, forming red tides [17], and be classified as Harmful Algal Blooms due to toxin production or contribution to anoxic conditions [18, 19]. We can therefore expect different impacts of dinoflagellate and diatom blooms on their surrounding communities.

Coastal ecosystems are composed of hundreds of organisms that interact with one another, forming complex networks of relationships [20, 21]. More recently, species interactions have gained increasing attention, as they can significantly influence community assembly, species response to climate change, and exacerbate anthropogenic pressures [22, 23]. One debated hypothesis suggests that while the environment selects for functions, biotic interactions select for species [24]. Interactions with viruses, bacteria, and eukaryotic parasites play a crucial role in phytoplankton dynamics [25–30]. Interactions within phytoplankton communities should also be considered. Several studies have highlighted the importance of allelopathy. Allelopathy can have deleterious effects and be considered as a weapon for interspecific competition, or have a positive effect on the whole community if the compounds act against “common enemies” [31, 32]. Competition can also arise among phytoplankton, and some dinoflagellates have developed adaptations such as vertical migrations [33] and mixotrophy [34] to outcompete others. Facilitation is also considered a very common strategy in marine systems [35]. The work of Picoche and Barraquand [36] predicts that phytoplanktonic inter-genus interactions are primarily positive, with competition occurring mainly within genera (strong self-regulation) rather than between different genera. As such, community composition and responses to perturbations are shaped by interactions. While certain interactions may favor the emergence of a bloom, it is likely that blooms themselves exert an influence on the community structure.

Detecting and studying interactions is a complex challenge for ecologists [37]. Association network analyses are commonly used and can be valuable for identifying associations between species, in particular for uncultivated microbes, through co-abundance correlations, which can be summarized using graph theory. Distinct community structures result in different graph topologies, which are useful for comparing community states and their evolution [29, 38, 39]. Some research has focused on the role of environmental conditions in shaping association networks [40–42] and on the importance of seasonality [43]. Association network analyses are also widely applied to investigate interactions between prokaryotes and bacteria, including during bloom events, using metagenomic approaches and over relatively short timeframes [30, 44].

The present study analyses the impact of blooms on phytoplankton diversity and ecological associations, focusing specifically on inter-genus phytoplankton associations. Using a 16-year time series covering the coastal waters of mainland France, derived from the French phytoplankton monitoring survey (REPHY), we addressed the following questions through temporal association networks. How do phytoplankton blooms affect the structure and diversity of associations within phytoplankton communities? Are specific types of inter-genus phytoplankton associations favored during a bloom? Do blooms of diatoms and dinoflagellates have distinct impacts on community structure? Addressing these questions is essential to enhance our understanding of bloom impacts on phytoplankton communities and to gain deeper insights into the differences between diatom and dinoflagellate bloom dynamics. To our knowledge, this is the first study to investigate phytoplankton associations across multiple regions and over a decadal timescale, in order to understand the effects of recurrent bloom events.

## Materials and methods

### Phytoplankton and environmental data

The REPHY monitoring survey has been collecting, fortnightly, information on the phytoplankton community through abundances associated with hydrology measurements at the surface since 1987 [45]. Sampling stations are distributed in coastal waters all around mainland France, and the sampling and analyses are conducted through a unified procedure [46]. The environmental variables used in this study were seawater temperature, salinity, turbidity, chlorophyll *a* concentration (chl*a*), dioxygen concentration, and nutrient concentration: nitrate plus nitrite, ammonia, silicate, and phosphate. Phytoplankton samples were analyzed following the Utermöhl method [47]: a 1 l water sample is fixed with Lugol’s solution before settling in a 10 ml sub-sample within an Utermöhl chamber. Phytoplanktonic cells with a diameter larger than 20 μm were counted and identified to the lowest taxonomic level. Smaller organisms can also be identified if they are known to be harmful. As a result, REPHY’s definition of phytoplankton is not strictly taxonomic and aligns more closely with microplankton *sensu stricto*. Since different experts identified the phytoplankton, we used the genus as the lowest taxonomic level to reduce identification bias.

**Figure 1:**
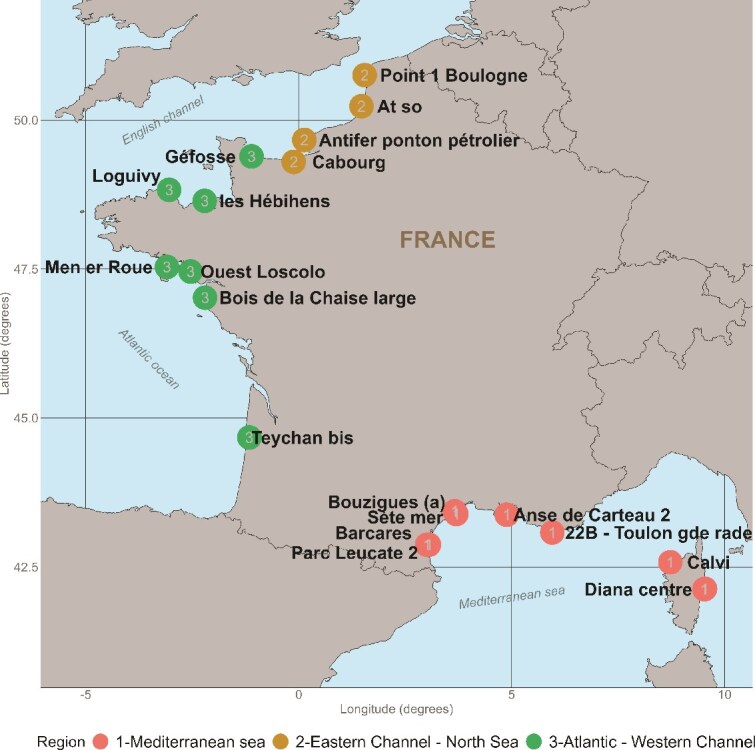
Map of the sampling sites used in this study. The REPHY sampling sites are coloured by region: Mediterranean Sea (1), Eastern Channel—North Sea (2), Atlantic—Western Channel (3).

From 195 sampling sites monitored since 1995, we selected 46 sampling stations for which the length of the chl*a* time series without a 2-month gap was longer than 5 years (except during the COVID-19 pandemic). These 46 sampling stations were used to regionalize the dataset (see below). Then, a second selection was conducted to retain 21 chl*a* time series with at least 15 years of data within the same period, i.e. from March 2007 to August 2022 (Fig. 1). Two stations showed an abrupt change in species richness, which we inferred to be due to a change in sampling analysis design. Consequently, these two stations were excluded from further analysis.

### Hydrology-based regionalization

Our stations cover all mainland French coastlines, which differ in biological and physicochemical ways [48, 49]. To study the broader geographical area beyond each individual station, a hydrology-based regionalization that considers the proximities between them was conducted. This approach enabled us to account for local characteristics that are not captured in the data.

The first step involved handling missing data. To implement this, we used the “EM-PCA” algorithm [50]. In the second step, a principal component analysis (PCA) was performed on all environmental variables. From this PCA, the median position across the dimensions for the 46 stations was calculated. The following steps were based on the methodology of Chavent *et al.* [51]. Briefly, an initial clustering was carried out using the median PCA positions on all dimensions; a second one was conducted on the spatial locations of the sampling stations using the same method. A geographical constraint weight was defined to maximize hydrological differences while maximizing the spatial constraints. We set this weight to 10% for the spatial constraint (see Supp. Mat. 1 for details). A composite distance matrix was generated and used for clustering. The optimal number of regions was determined using the silhouette index.

### Global and temporal association networks

The community structure of phytoplankton was assessed through their biotic associations proxied by association networks. Association networks are based on correlations between taxa abundances, where nodes represent taxa and edges represent associations between pairs of taxa. This approach assumes that correlation implies association. Fourteen graph metrics were computed to capture the main features of the networks (Table 1).

**Table 1:** Definition and abbreviation of the graph metrics used in this study.

Abbreviation	Definition
N_nodes	Number of nodes: *equal to species richness.*
N_edges	Number of edges: *number of associations.*
Dens	Density: corresponds to N_nodes/N_edges.
Avg_degree	Average degree: average number of edges per node.
Diss	Dissimilarity: quantifies how well the nodes are connected to each other based on the number of links between them.
Trans	Transitivity: corresponds to average local connectivity and quantifies how well the adjacent nodes are connected to each other.
C_tance	Connectance: percentage of realized edges out of all possible edges. *Related to association specificity.*
Avg_edge_bet	Average edge betweenness: quantify the average importance of edges; it is calculated based on the number of nodes that rely on this edge to connect to the hubs (nodes establishing more links with other nodes, which themselves are establishing more).
Avg_p_length	Average path length: the length of a link is shorter if the correlation between nodes is strong. *Opposite to average associations strength*.
Adhes	Adhesion: number of edges to delete to disconnect the network. *Related to the sensitivity of the network to the existence of association.*
Nat_connect	Natural connectivity: number of nodes to delete to disconnect the network. *Related to the sensibility of the network to taxa composition.*
Mod	Modularity: a set of nodes that have more edges with each other than with the others. *Quantify how the network is organized into sub-communities*.
N_clust	Number of clusters: number of clusters defined by the fast greedy clustering method. *Rely on identifying sub-communities.*
Assort	Assortativity: tendency of nodes to connect to nodes with the same number of relationships with other nodes. *If assortativity is positive, this means that highly connected taxa, often generalists that interact with many other species, tend to connect with each other, and similarly for taxa with few connections, specialists.*

Italic text provides an ecological interpretation when possible.

A global association network was performed for each region using phytoplankton abundances. Absent taxa were given an abundance of zero. Only taxa present in more than 30 dates were selected in order to exclude rare taxa and avoid false positive associations. The Spearman correlation coefficient was used to calculate the correlation between taxa abundances. Only significant positive correlations, whose *P*-value was corrected by the Benjamini–Hochberg method [52], and significantly different from zero, were preserved, using the NetCoMi R package [53] because negative associations often reflect niche differentiation and are more difficult to detect than positive associations [43]. Negative associations were considered only for the analysis of association types (see below).

One of the main limitations of global association networks is their static nature, as they are constructed using the entire time series across all stations within a given region. To deal with temporal dynamics, temporal networks were extracted from the global network. The process involves extracting nodes that correspond to the taxa present at a specific date and station. By doing so, we also kept the associations between pairs of nodes. That means that we have one network per sampling date for each station, allowing us to calculate graph metrics for each temporal network as well as for the global network. To reduce the dimensionality of the different graph metrics, a PCA was performed.

### Network compositions

To analyze association networks, we first examined their taxonomic composition, determined by the number of nodes belonging to each taxonomic class. We focused on three classes: diatoms (Bacillariophyceae), dinoflagellates (Dinophyceae), and others (“Other phyla”). We also analyzed the composition of edges, which provides insights into the “association types” within the network. We focused on association between diatoms, between dinoflagellates, between diatoms and dinoflagellates, and any association involving a taxon from another phyla.

The analysis of the association networks’ compositions provides the proportion of each association type. To determine whether these proportions result from ecological processes rather than basic taxonomic composition, randomization tests were performed. If the proportions were solely due to composition, we would expect random associations to yield similar proportions. However, while it is well-established that associations themselves are influenced by ecological processes, it is less clear whether this also applies to the association type proportions. To assess this, we took the association matrix for each region. Simple random permutations were performed; by doing so, species richness and the number of associations remained the same. This process allowed us to calculate the proportion of each association type in a “null” association network. 10 000 null association networks were generated for each region. We compared the null distribution of the proportion of an association type to the observed proportion of that association type in the global network by calculating *P*-values. Because we obtained a *P*-value for each association type, a global *P*-value was calculated based on Fisher’s correction for each region.

### Diversity indices

Three diversity indices were calculated from the abundance data for each sampling day. The Shannon index is a biodiversity index that considers species richness and equitability [54]. The Pielou index is used to estimate how equitable the abundances of species in a community are [55]. The Berger–Parker index corresponds to the proportion of the most abundant taxon to estimate its dominance [56].

### Bloom detection and characterization

The definition of bloom for our study is based on the ICES definition considering the comments of Isles and Pomati [6]: a bloom is a deviation from the “normal” seasonal cycle of phytoplankton biomass. Here, we used chl*a* as a proxy of biomass. The time series for each station was processed equally. Chl*a* time series were regularized using linear interpolation. The seasonal component was subtracted by seasonal-trend decomposition by Loess [57] assuming that the seasonal component is identical over the years. Phytoplankton blooms were identified from the deseasonalized chl*a* time series using outlier detection through the median absolute deviation [58]. The threshold coefficients described in Leys *et al.* [58] were chosen to be more objective.

For each bloom detected, we assumed that the most abundant taxa were responsible for it. We determined the first previous sampling date as the “before” moment and the first following sampling date as the “after” moment of the bloom. This choice was made because of the fortnightly sampling. We conducted sensitivity analyses to restrict the time window before and after the bloom to varying durations, but these adjustments did not affect the results. If there are successive dates that are determined as blooms and if the class remains the same (e.g. from *Chaetoceros* to *Skeletonema* blooms, two diatoms), we consider only one “before” and “after” moments. If there are successive dates that are determined as blooms but the class changes (e.g. from diatom to dinoflagellate bloom), we consider the first bloom with a “before” moment and the second with an “after” moment. If a sampling date is both an “after” and a “before” we excluded it from bloom analysis.

To estimate whether a bloom means a higher dominance of a particular taxon, we compared the Berger–Parker index for bloom and “non-bloom” dates. For this analysis, 100 random “non-bloom” dates were selected, controlling for the season (spring and summer are overrepresented within bloom dates). We compared this across all regions and by region, using a Wilcoxon test.

### Statistical analysis and software

Statistical significance was defined as *P* < .05 after correction for multiple testing. Kruskal–Wallis tests were used to assess differences between regions, types of blooms, and bloom moments. If the test was significant, a Dunn test with Benjamini–Hochberg’s method for *P*-value correction was done. All analyses were performed using R 4.4.2 [59]. Further details are available in the Data availability section.

## Results

### Phytoplankton associations and diversity in French coastal waters

Three regions were obtained from the regionalization process (Fig. 1). The first region corresponds to the Mediterranean Sea, the second to the Eastern Channel—North Sea (hereafter Eastern Channel-NS), and the third to the Atlantic Ocean—Western Channel (Atlantic-WC).

**Figure 2:**
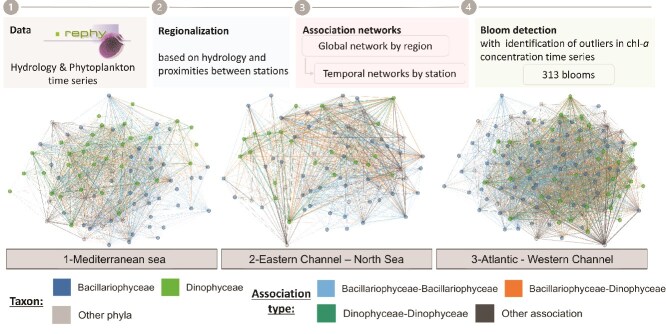
Average relative abundance by month and region from 2007 to 2022 for the four most abundant taxa by season and region.

The mean relative abundance of diatoms was 72.10% in the Mediterranean Sea and 75% for the other two regions. Dinoflagellates are the second most dominant class, with a mean proportion of 17.69% in the Mediterranean Sea and ~6% in the other regions. The three regions differed in terms of phytoplankton composition at the genus level (Fig. 2). For example, there was a higher relative abundance of *Skeletonema* in the Mediterranean Sea, *Phaeocystis* in the Eastern Channel-NS, and *Asterionnellopsis* in the Atlantic-WC region. Analyzing the temporal pattern of composition revealed that extreme dominance of the community by one taxon can display a well-established seasonality (for instance, *Phaeocystis* dominance occurs consistently around March in the Eastern Channel-NS) or emerge suddenly (for instance, high dominance of *Chrysochromulina* in June 2012, *Pseudo-nitzschia* in June 2015, or *Azadinium* in February 2020 in the Atlantic-WC).

**Figure 3:**
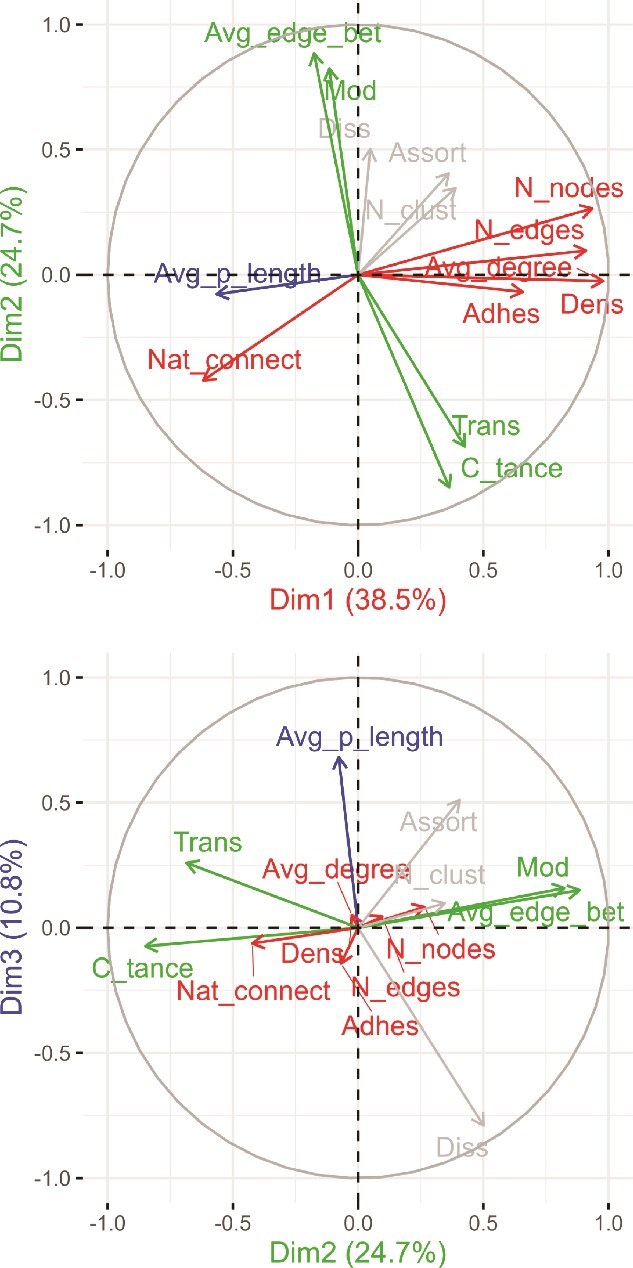
PCA based on graph metrics. The variance explained by each dimension is indicated in the axis labels. Graph metrics were calculated for all temporal networks across all regions. See Table 1 for the definition of each abbreviation.

The PCA based on the graph metrics allows us to identify the association network structures and also how graph metrics are related to each other (Fig. 3). The first three dimensions of the PCA explained 74% of the total variance in graph metrics (38.5%, 24.7%, and 10.8%, respectively). The first dimension (Dim1) primarily captures the variations in species richness and the robustness of the network. Along this dimension, the number of nodes, edges, density, and adhesion are positively correlated with each other. The second dimension (Dim2) can be interpreted in terms of modularity and association selectivity. Along Dim2, modularity and dissimilarity are positively correlated with each other, while connectance and transitivity are negatively correlated with Dim2. The third dimension is mainly positively correlated with average path length, meaning that high Dim3 values indicate a low average association strength.

**Figure 4:**
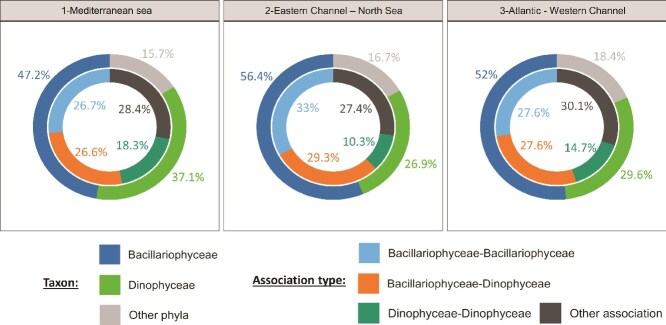
Global association networks composition by region. The outer circle represents the relative number of each taxon in the global network, *Bacillariophyceae, Dinophyceae*, or other phyla. The inner circle indicates the relative proportion of each association type. Associations between *Bacillariophyceae*, between *Dinophyceae*, between *Bacillariophyceae* and *Dinophyceae*, or other types of associations.

Regional global association networks differed by structure and association types (Figs S1 and S2, Fig. 4). The Mediterranean Sea region has the lowest species richness and is more sensitive to the extirpation of a single species (Nat_connect) (Fig. S3). Conversely, the Atlantic-WC region has the most robust network and was significantly more organized into sub-communities with high association specificity than the Eastern Channel-NS, which was, in turn, more structured than the Mediterranean Sea region. The average association strength was significantly lower in the Eastern Channel-NS region, while it was similar between the other two regions (Fig. S3). Most associations were positive: 75.97%, 63.76%, and 64.46% in the Mediterranean, Eastern Channel-NS, and Atlantic-WC networks, respectively (Fig. S4). In accordance with phytoplankton counts, diatoms represented the largest proportion of taxa in the global networks, followed by dinoflagellates (Fig. 4). Consistently, associations between diatoms were the most frequent across all regions, followed by associations between diatoms and dinoflagellates, except in the Atlantic-WC region where 31.6% of the links were related to other types of associations (Fig. 4). The patterns remains regarding negative associations (Fig. S4). It is important to remember that this applies to the global networks, and there is a great temporal variability (Fig. S2).

Importantly, the proportion of association types results from ecological processes and not from random processes. In all regions, the association type proportions significantly differed from a random distribution of associations: the randomization test had a *P*-value <2.2e-16 in the Mediterranean Sea, a *P*-value = 2.34e-10 in the Eastern Channel-NS, and a *P*-value <2.2e-16 in the Atlantic-WC (Fig. S5).

### Bloom detection

Our method for bloom detection identified 125 blooms in the Mediterranean Sea, 67 in the Eastern Channel-NS, and 121 in the Atlantic-WC region. Two-thirds of all detected blooms were blooms of diatoms (Table 2; Fig. S6). Cryptophytes in the Mediterranean Sea, haptophytes in the Eastern Channel-NS, and dinoflagellates in the Atlantic-WC region were the second most frequent classes in terms of bloom occurrence. A total of 13 dinoflagellate blooms were detected in the Mediterranean Sea, 5 in the Eastern Channel-NS, and 10 in the Atlantic-WC region. Additionally, 14 haptophyte blooms related to *Phaeocystis* were identified in the Eastern Channel-NS. This small number of blooms may limit the statistical power to evaluate their impacts (Table S1). The genera responsible for blooms differed between regions (Table 2). For example, 10 *Cylindrotheca* blooms were detected in the Mediterranean Sea, of which 9 were in coastal lagoons, but none were in the other regions. *Prorocentrum* blooms were the most detected dinoflagellate blooms in the Mediterranean Sea, whereas it was *Lepidodinium* in the two other regions.

**Table 2:** Blooms detected by region, categorized by class and genus.

Region	Class/genus	Number of blooms
1-Mediterranean Sea	***Bacillariophyceae***	**94**
	*Chaetoceros*	*40*
	*Pseudo-nitzschia*	*19*
	*Skeletonema*	*11*
	*Cylindrotheca*	*10*
	*Leptocylindrus*	*7*
	*Dactyliosolen*	*2*
	*Naviculaceae*	*1*
	*Rhizosolenia*	*1*
	*Nitzschia*	*1*
	*Cerataulina*	*1*
	*Thalassionema*	*1*
	***Cryptophyceae***	***16***
	***Dinophyceae***	***13***
	*Prorocentrum*	*7*
	*Gymnodinium*	*2*
	*Gyrodinium*	*2*
	*Protoperidinium*	*1*
	*Gonyaulax*	*1*
	***Dictyophyceae***	***2***
	*Dictyocha*	*1*
	*Dictyophyceae*	*1*
2-Eastern Channel – North Sea	***Bacillariophyceae***	***47***
	*Chaetoceros*	*25*
	*Asterionellopsis*	*7*
	*Leptocylindrus*	*6*
	*Skeletonema*	*3*
	*Guinardia*	*3*
	*Pseudo-nitzschia*	*1*
	*Thalassiosira*	*1*
	*Lauderia*	*1*
	***Haptophyta***	***14***
	*Phaeocystis*	*14*
	***Dinophyceae***	***5***
	*Lepidodinium*	*2*
	*Akashiwo*	*1*
	*Gymnodinium*	*1*
	*Prorocentrum*	*1*
	***Cryptophyceae***	***1***
3-Atantic – Western Channel	***Bacillariophyceae***	**106**
	*Guinardia*	16
	*Dactyliosolen*	14
	*Skeletonema*	13
	*Thalassiosira*	12
	*Leptocylindrus*	11
	*Chaetoceros*	10
	*Asterionnellopsis*	8
	*Cerataulina*	5
	*Rhizosolenia*	5
	*Pseudo-nitzschia*	4
	*Chaetocerotaceae*	3
	*Lauderia*	3
	*Plagiogramma*	1
	*Lithodesmium*	1
	***Dinophyceae***	**10**
	*Lepidodinium*	7
	*Gymnodiniales*	1
	*Lingulodinium*	1
	*Prorocentrum*	1
	***Haptophyta***	**2**
	*Phaeocystis*	2
	***Cryptophyceae***	**2**
	*Cryptophyceae*	1
	*Cryptomonadales*	1
	***Ciliophora***	**1**
	*Mesodinium*	1

### Bloom’s impact on diversity, community structure, and associations

**Figure 5:**
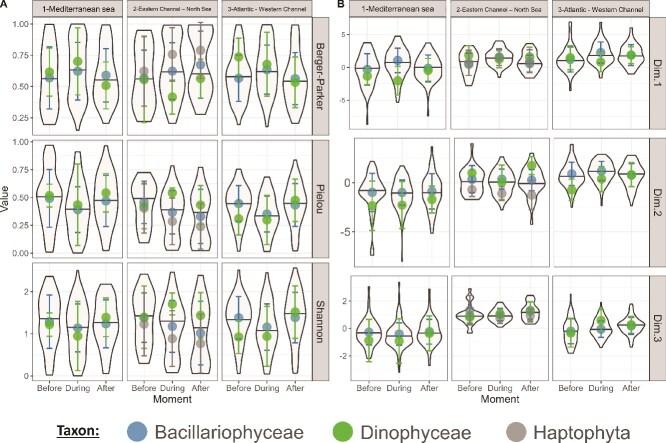
Diversity indices (A) and PCA dimensions (B) before, during, and after a bloom for each region. The violin plot shows the distribution of values for diatom and dinoflagellate blooms, as well as Eastern Channel’s haptophyte blooms. The black line represents the mean value. Points indicate the mean value for *Bacillariophyceae, Dinophyceae*, and *Haptophyta* blooms. Error bars represent the standard deviation.

On average, the Shannon and Pielou indices were lower during dinoflagellate or diatom blooms, except in the Eastern Channel-NS region, which exhibited a different pattern (Fig. 5A). The Berger–Parker index generally increased during blooms. Coherently, when comparing bloom to non-bloom samples, our results indicate a higher Berger–Parker index (12%), which seems to be mainly driven by the Mediterranean region (Fig. S7). Additionally, no correlation between chl*a* concentration and the Berger-Parker index (Spearman’s correlation) was found.

Blooms also impact the structure of association networks (Fig. 5B). Our results revealed an increase in the Dim1 PCA coordinates during diatom and haptophyte blooms, with varying amplitudes depending on the region. In contrast, during dinoflagellate blooms, Dim1 values decreased. These findings suggest that diatom and haptophyte blooms are associated with an increase in species richness (N_nodes) and network robustness (Adhesion), whereas dinoflagellate blooms have the opposite effect. Blooms’ impacts on modularity and association strength appeared minimal.

**Figure 6:**
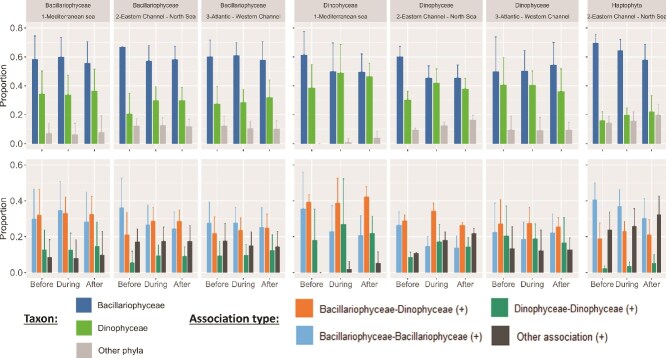
Composition of positive association networks before, during, and after *Bacillariophyceae, Dinophyceae*, or *Haptophyta* blooms for each region, expressed as a proportion of all associations (positive and negative). The top bar plots show the average proportion of each taxon on the association networks (*Bacillariophyceae, Dinophyceae*, other phyla). The bottom bar plots display the average proportion of each association type (between *Bacillariophyceae*, between *Bacillariophyceae* and *Dinophyceae*, between *Dinophyceae*, and other types of associations). Error bars represent the standard deviation.

Blooms maintain the proportions of taxonomic composition in the networks (Fig. 6). Our results indicate that during blooms, the relative proportions of taxa in the association networks remained stable. While species richness changed (Fig. 5B), no taxonomic class exhibited a significant increase in proportion at any moment of the bloom (Fig. 6). This indicates a stability in the taxonomic composition of association networks. Although differences emerged depending on the bloom taxonomic class (e.g. proportion of dinoflagellates was higher in whatever the phase of dinoflagellate blooms compared to other bloom types), this is probably due to different seasonal preferences between classes.

A regional similarity was observed for each bloom type, though it was less consistent for dinoflagellate blooms, especially for the “Other association” type in the Mediterranean. Examining the networks association types revealed that, like taxonomic composition, proportions remained relatively stable across bloom phases, and regions, while the proportion of association types varied depending on the bloom taxonomic class (Fig. 6). Around dinoflagellate blooms, networks contained more associations involving dinoflagellates than other bloom types, while around haptophyte blooms, “Other association” types were more prevalent. The same pattern is observed when focusing on negative associations. Negative associations remain very low in proportion as compared to positive associations and also stable across bloom phases and region (Fig. S8).

Overall, these findings highlight that bloom impacts depend on the taxonomic nature of the dominant taxon, but also reveal similarities across regions regarding a general stability of association networks in taxonomic and association-type composition across different bloom phases.

## Discussion

### Phytoplankton communities and association structures in French coastal waters

Our regionalization process identified three coastal regions in France. It is well known that the Mediterranean Sea is a distinct ecosystem compared to the Atlantic Ocean and the English Channel [49, 60], accentuated in our dataset by the prevalence of coastal lagoons in the Mediterranean region. This regionalization may seem trivial, but we argue that it is necessary to differentiate regions objectively and without prior assumptions. The fact that the Western Channel stations are more similar to the Atlantic stations supports this approach.

The three regions presented distinct communities, consistent with their different local environments and ecosystem functioning. The Shannon index was lower in the Mediterranean Sea, which could be partly explained by species smaller than 20 μm (e.g. cyanobacteria) that are not systematically counted [61]. Diatoms were found to be dominant in phytoplankton communities across all regions, which is consistent with previous findings [62]. In all regions, most associations were positive, a result also observed in other studies [13, 28, 43]. This supports the idea that inter-genus associations are primarily positive as argued by Picoche and Barraquand [36]. Different association structures were found between regions, reflecting distinct communities. The Atlantic-WC region appears to have the highest species richness and to be more organized into sub-communities. However, this may be influenced by the higher number of sampling dates in this region and its broader latitudinal range, potentially introducing biases and apparently increasing the number of detected associations (i.e. sampling effect; [63, 64]).

A key finding of our study is that association type proportions result from ecological processes, and therefore they must be further investigated. This implies association type proportions likely play a key role in community equilibrium and responses to perturbations. The diversity of interaction types between species remains understudied, as they are difficult to capture, predict, and validate. Previous works [65, 66] demonstrated that the balance of interaction types plays an important role in community dynamics. Thus, the diversity and proportion of interaction types may also play a crucial role in community responses to perturbations and overall ecosystem functioning.

### Blooms’ impact on diversity

The method used for bloom detection has several advantages: it is simple, auto-adaptive to the study site, and does not rely on prior assumptions. However, defining the taxa responsible for the bloom as the most abundant one may be a simplistic and reductionist approach, as some blooms are mixed events of multiple taxa, and some extreme biomass can be the result of taxa that are underestimated in REPHY (i.e. nano- and pico-phytoplankton). Monospecific and polyspecific blooms may have different origins and effects on the community structure [67]. Defining the phases of a bloom as the first previous and following dates allows for analysis of bloom dynamics without considering temporal changes in abiotic factors. However, we cannot exclude the influence of abiotic factors, but we consider that their influence is reduced as much as it can be considering the time span between dates (Fig. S9). This also aligns with the difficulty in understanding the abiotic factors favoring blooms but the objective here is to focus on biotic factors. Blooms occurred in all seasons: this supports the idea that studying blooms all year long (and not only during spring) is essential for a better understanding of phytoplankton ecology, as previously highlighted by Smayda [3]. We detected a relatively large number of blooms, with most blooms being dominated by diatoms, as generally observed in coastal waters [15, 62]. Dinoflagellate blooms may be less frequent, shorter, and/or more localized, and therefore less likely to be sampled by regular monitoring programs than blooms of diatoms [18].

A key result is that species richness, measured by the number of network nodes, increases during diatom and haptophyte blooms but decreases during dinoflagellate ones. It suggests different community responses during these blooms. Several hypotheses can explain lower species richness during dinoflagellate blooms. Some dinoflagellates have deleterious effects on other phytoplankton, excluding competitors through allelopathy [31, 68] or predation for mixotrophy [69, 70]. Beyond direct interactions, environmental conditions favoring their blooms may be less suitable for diverse phytoplankton taxa. For instance, in heavily stratified waters, intense vertical migrations allow dinoflagellates to exploit deep, nutrient-rich layers inaccessible to their competitors [33]. This aligns with the observation that dinoflagellate blooms occur more sporadically and ephemerally than diatom blooms, with more restrictive ecological niches [16]. Contrary to dinoflagellate blooms, diatom and *Phaeocystis* blooms showed higher species richness than before and after the bloom. During *Phaeocystis* blooms, cells can agglomerate and form mucus, which acts as a defense against grazers [71, 72]. Can the mucus-covered colonies of *Phaeocystis* act as an “umbrella” and protect other genera from grazers, enhancing species richness? Diatom blooms may, in turn, exhibit greater species richness due to the facilitative actions of taxa and better self-regulation, allowing them to coexist. However, all these hypotheses remain to be tested, e.g. using mesocosm experiments, as we cannot directly assess the underlying processes. Some authors [73, 74] have focused on determining the dominant processes operating in microbial communities, including during bloom phases, namely deterministic and stochastic processes, using network-based approaches. They showed that both deterministic and stochastic processes can explain microbial community succession, depending on the bloom phase and the prevailing abiotic conditions. The abiotic conditions that favor each type of bloom remain a key challenge in marine ecology [75].

Blooms also impact diversity. The Berger–Parker index increased during blooms, regardless of chl*a* levels, highlighting the limits of some bloom definitions based on abundance thresholds. This pattern suggests dominance by a single taxon, likely due to competitive exclusion mechanisms such as allelopathy, mixotrophy, nutrient affinity, or grazing resistance [31, 34, 69, 76–78]. In two regions (Mediterranean and Atlantic), the Shannon and Piélou indices decreased during blooms, reflecting uneven species abundances and a community dominance, as confirmed by the Berger–Parker index. However, in the Eastern Channel-NS region, the diversity indices have a different pattern, likely due to specific bloom-forming taxa, abiotic environment, or community structure. These diversity changes were not linked to species richness, indicating that blooms reorganize the abundances rather than change species presence. This is illustrated by changes in species richness and Shannon index. During diatom blooms, species richness increased while Shannon index decreased because of the decline in evenness. This highlights the limitation of relying on a single alpha diversity metric and supports the use of multiple metrics for a better interpretation of biodiversity patterns.

### Blooms impact on community structure and associations

The impact of blooms on community structure is more difficult to disentangle, as our results do not give a clear signal regarding graph metrics. Additional higher-resolution data could reveal the effect of blooms on community structure and associations. In that case, the high variability observed would indicate that these effects cannot be adequately studied with a classification as broad as all diatom or dinoflagellate blooms, regardless of season, and separated into three regions. This issue brings us back to a fundamental challenge in ecology: the need to group elements into broader categories to understand certain processes, even when some phenomena may not be fully captured within these broader groupings [79, 80].

### The importance of equilibria in phytoplankton blooms

A key result is that broad taxonomic composition and types of association remain unchanged during blooms, regardless of bloom type or region, suggesting that no association type is favored during a bloom, this also holds for negative associations. A surprising finding is that the composition and proportions of associations are highly similar across regions. Our randomization test confirmed that association type proportions are shaped by ecological processes and not merely a reflection of the proportion of each taxonomic class (Fig. S5). The fact that species richness changes during blooms makes this result even more unexpected. Blooms may not represent a complete renewal of the community but rather a reorganization of abundances through self-regulation and compensatory mechanisms. Jochimsen *et al.* [81] and Gonzalez and Loreau [82] demonstrated that compensatory dynamics and interaction types are key factors contributing to phytoplankton resilience and stability. This aligns with the idea that association type proportions are essential for the resilience and functioning of phytoplankton communities through regulatory mechanisms. This also supports the idea that competition primarily occurs within genera [36]. The community persisting during blooms may consist of taxa with high functional redundancy relative to the “non-bloom” community. In addition, functional redundancy is known to enhance community resilience [83–85]. Taxonomic classification overlaps significantly with functional classification, further supporting this argument [86, 87].

Exploring the notion of a core community could be particularly insightful. A core community consists of taxa that consistently occur, are locally abundant, and regionally common, in contrast to satellite or transient communities, which are sparse and occur less frequently [88, 89]. Core species are hypothesized to be better adapted to local environmental conditions than transient species, and therefore, more likely to exhibit traits indicating environmental tolerance [90]. The core community may remain relatively stable, as observed in our analysis, while transient species are more affected by blooms. Consequently, the core community could exhibit distinct characteristics in terms of modularity, species richness, and average association strength. Xue *et al.* [91] demonstrated, through network analysis, that core communities exhibit more stable structures against perturbations than satellite communities. Blooms are likely not disruptive enough to impact this core community, which ensures resilience and a return to the previous functional state. However, methodological challenges and ongoing debates persist regarding the validity of this classification [92, 93]. Deutschmann *et al*. [94] observed that no association was consistently present in the marine microbial network they studied. The authors question the notion of a core interconnected microbiome, instead suggesting that a small core of highly recurrent associations, surrounded by a large set of transient or seasonal associations, structures the entire community. Consequently, the functional stability of marine ecosystems relies on redundancy and seasonal recurrence rather than on conserved interactions. The analyses of keystone species within networks can provide some insightful elements [29, 76].

Overall, the proportions differ between diatom, dinoflagellate, and haptophyte blooms. This highlights that when diatoms bloom, there are proportionally more diatoms both before and after the bloom, along with a higher number of associations involving diatoms, likewise during dinoflagellate blooms: more dinoflagellates and more associations involving them. This result suggests that the environmental context and abiotic forcing play a key role in shaping both community composition and the proportions of association types, more so than the influence of blooms themselves.

### Association networks to study phytoplankton communities

Association network analysis is useful to estimate associations between taxa. Here, “association” does not necessarily mean “interaction” as we cannot disentangle both nor define the type of interaction. Different interactions can result in the same signal (e.g., facilitative or mutualistic). It is likely that correlations do not reflect only biotic interactions but also largely overlapping niches. Another limitation of this approach is that it provides a global association coefficient. This implies that we cannot consider changes in the signs or strength of the association between two given taxa. Indeed, species may modify their interactions in the presence of others or in different environmental conditions [37]. This modification can have a great impact on the overall ecosystem structure and functioning [95]. Previous modeling studies demonstrated that interaction strength variability is a key component for ecosystem stability and species coexistence because species interactions can be modulated by biotic or abiotic changes [96, 97]. Despite such limitations, the relevance of using association networks to study phytoplankton interactions has been demonstrated: such approach was proven to be able to recover known interactions as well as discover new ones [28, 44, 98].

Our results provide an understanding of what association networks can capture in terms of community ecology. The number of nodes, edges, and adhesion covary. This can be explained because the number of edges follows a quadratic function with the number of nodes [98]. Modularity increases when connectance decreases, meaning that the community is more organized into sub-communities when association specificity is high. It is interesting to note that species richness is not related to sub-community organization, indicating that an additional taxon can be well integrated into a sub-community. Studies have found a positive correlation between robustness and connectance, which we did not recover in our analysis [99, 100]. Another important graph metric is the average association strength (Dim3). We initially hypothesized that weak association strength could indicate a more sensitive community because taxa are less influenced by each other. However, the work of Allesina and Tang [101] supports the idea that stability is achieved by decreasing the average interaction strength, thereby lowering complexity. Accordingly, Carpentier *et al.* [102] demonstrated a trade-off between robustness and stability in biological networks. Other work from Thebault and Fontaine [103] concluded that stability of trophic networks is higher in modular and weakly connected architectures. Altogether, it appears that community stability is difficult to estimate through graph metrics in our analysis, since robustness, modularity, and interaction strength are not linked. Integrating the analysis of keystone species can provide insightful information to disentangle the link between network structure and community stability [104].

## Conclusion

Through the analysis of 16 years of phytoplankton abundance data using association networks, our study highlighted the influence of blooms on microphytoplankton community structure, species richness, and association dynamics. We show that diatoms and dinoflagellates reduce diversity indices, likely due to a reduction in evenness rather than a change in species richness by competitive exclusion. On the other hand, diatom and haptophyte blooms increase species richness, whereas dinoflagellate blooms lead to their decline. Despite these variations, taxonomic composition and association type proportions remain mostly unchanged during blooms. This suggests strong regulatory mechanisms within phytoplankton communities and a role of these proportions in community functioning. In addition, there seems to be no specific types of inter- or intra-class phytoplankton associations favored during a bloom. Diatoms and haptophytes respond to bloom conditions in a more similar way, whereas dinoflagellates generally show opposite patterns. This supports the idea of distinct mechanisms initiating and supporting these types of blooms. Future studies should refine these findings by integrating keystone species analysis, functional classifications, exploring the role of sub- or core-communities in community stability, and testing precise ecological processes. Our work confirms the importance of studying biological interactions to understand bloom dynamics at a community level.

## Supplementary Material

Supplementary_M_Diatoms_vs_dinoflagellates_vRview_final_ycag174

## Data Availability

The raw data that support the findings of this study were provided by IFREMER and are available from SEANOE, at https://doi.org/10.17882/47248. All R scripts and generated data used in this study are available from the Github repository, at github.com/J-YDi/Diatoms-vs-Dinoflagellates and from zenodo at 10.5281/zenodo.18785609.

## References

[1] Karlusich JJP, Ibarbalz FM, Bowler C. Exploration of marine phytoplankton: from their historical appreciation to the omics era. *J Plankton Res* 2020;42:595–612. 10.1093/plankt/fbaa049

[2] Parker M . Exceptional marine blooms and their implications for fisheries. *ICES Coop Res Rep* 1983;124:25–8.

[3] Smayda TJ . What is a bloom? A commentary. *Limnol Oceanogr* 1997;42:1132–6. 10.4319/lo.1997.42.5_part_2.1132

[4] Tett P . The ecophysiology of exceptional blooms. *Rapp P-V Reun Cons Int Explor Mer* 1987;187:47–60.

[5] Kim HG, Park JS, Lee SG. et al. Population cell volume and carbon content in monospecific dinoflagellate blooms. In: Smayda T., Shimizu Y. (eds.), Toxic Phytoplankton Blooms in the Sea. Amsterdam: Elsevier Sci. Publ, 1993, 769–73.

[6] Isles PDF, Pomati F. An operational framework for defining and forecasting phytoplankton blooms. *Front Ecol Environ* 2021;19:443–50. 10.1002/fee.2376

[7] Sverdrup HU . On conditions for the vernal blooming of phytoplankton. *ICES J Mar Sci* 1953;18:287–95. 10.1093/icesjms/18.3.287

[8] Huisman J, Van Oostveen P, Weissing FJ. Critical depth and critical turbulence: two different mechanisms for the development of phytoplankton blooms. *Limnol Oceanogr* 1999;44:1781–7. 10.4319/lo.1999.44.7.1781

[9] Behrenfeld MJ, Boss ES. Resurrecting the ecological underpinnings of ocean plankton blooms. *Ann Rev Mar Sci* 2014;6:167–94. 10.1146/annurev-marine-052913-021325 24079309

[10] Behrenfeld MJ, Boss ES. Student’s tutorial on bloom hypotheses in the context of phytoplankton annual cycles. *Glob Chang Biol* 2018;24:55–77. 10.1111/gcb.1385828787760 PMC5763361

[11] Behrenfeld MJ . Abandoning Sverdrup’s critical depth hypothesis on phytoplankton blooms. *Ecology* 2010;91:977–89. 10.1890/09-1207.1 20462113

[12] Behrenfeld MJ, Doney SC, Lima I. et al. Annual cycles of ecological disturbance and recovery underlying the subarctic Atlantic spring plankton bloom. *Global Biogeochem Cycles* 2013;27:526–40. 10.1002/gbc.20050

[13] Tan S, Zhou J, Zhu X. et al. An association network analysis among microeukaryotes and bacterioplankton reveals algal bloom dynamics. *J Phycol* 2015;51:120–32. 10.1111/jpy.12259 26986263

[14] Rinta-Kanto JM, Sun S, Sharma S. et al. Bacterial community transcription patterns during a marine phytoplankton bloom. *Environ Microbiol* 2012;14:228–39. 10.1111/j.1462-2920.2011.02602.x 21985473

[15] Carstensen J, Klais R, Cloern JE. Phytoplankton blooms in estuarine and coastal waters: seasonal patterns and key species. *Estuar Coast Shelf Sci* 2015;162:98–109. 10.1016/j.ecss.2015.05.005

[16] Smayda TJ, Reynolds CS. Strategies of marine dinoflagellate survival and some rules of assembly. *J Sea Res* 2003;49:95–106. 10.1016/S1385-1101(02)00219-8

[17] Gernez P, Zoffoli ML, Lacour T. et al. The many shades of red tides: Sentinel-2 optical types of highly-concentrated harmful algal blooms. *Remote Sens Environ* 2023;287:113486. 10.1016/j.rse.2023.113486

[18] Roux P, Siano R, Souchu P. et al. Spatio-temporal dynamics and biogeochemical properties of green seawater discolorations caused by the marine dinoflagellate Lepidodinium chlorophorum along southern Brittany coast. *Estuar Coast Shelf Sci* 2022;275:107950. 10.1016/j.ecss.2022.107950

[19] Anderson DM . HABs in a changing world: a perspective on harmful algal blooms, their impacts, and research and management in a dynamic era of climactic and environmental change. *Harmful Algae* 2012;17:3–17. PMC4667985 26640829

[20] Stachowicz JJ . Mutualism, facilitation, and the structure of ecological communities. *BioScience* 2001;51:235–46. 10.1641/0006-3568(2001)051[0235:MFATSO]2.0.CO;2

[21] Lang JM, Benbow ME. Species interactions and competition. *Nat Educ Knowl* 2013;4:8.

[22] Gilman SE, Urban MC, Tewksbury J. et al. A framework for community interactions under climate change. *Trends Ecol Evol* 2010;25:325–31. 10.1016/j.tree.2010.03.002 20392517

[23] Beauchesne D, Cazelles K, Daigle RM. et al. Ecological interactions amplify cumulative effects in marine ecosystems. *Sci Adv* 2025;11:eadp9315. 10.1126/sciadv.adp931539854468 PMC11759004

[24] Ramond P, Sourisseau M, Simon N. et al. Coupling between taxonomic and functional diversity in protistan coastal communities. *Environ Microbiol* 2019;21:730–49. 10.1111/1462-2920.14537 30672084

[25] Vincent F, Sheyn U, Porat Z. et al. Visualizing active viral infection reveals diverse cell fates in synchronized algal bloom demise. *Proc Natl Acad Sci U S A* 2021;118:e2021586118. 10.1073/pnas.202158611833707211 PMC7980383

[26] Cirri E, Pohnert G. Algae–bacteria interactions that balance the planktonic microbiome. *New Phytol* 2019;223:100–6. 10.1111/nph.15765 30825329

[27] Chambouvet A, Morin P, Marie D. et al. Control of toxic marine dinoflagellate blooms by serial parasitic killers. *Science* 2008;322:1254–7. 10.1126/science.1164387 19023082

[28] Lima-Mendez G, Faust K, Henry N. et al. Determinants of community structure in the global plankton interactome. *Science* 2015;348:1262073. 10.1126/science.1262073 25999517

[29] Sun P, Huang X, Wang Y. et al. Protistan-bacterial microbiota exhibit stronger species sorting and greater network connectivity offshore than nearshore across a coast-to-basin continuum. *mSystems* 2021;6:e0010021–1. 10.1128/msystems.00100-2134636671 PMC8510552

[30] Camarena-Gómez MT, Ruiz-González C, Piiparinen J. et al. Bacterioplankton dynamics driven by interannual and spatial variation in diatom and dinoflagellate spring bloom communities in the Baltic Sea. *Limnol Oceanogr* 2021;66:255–71. 10.1002/lno.11601

[31] Kubanek J, Hicks MK, Naar J. et al. Does the red tide dinoflagellate Karenia brevis use allelopathy to outcompete other phytoplankton? *Limnol Oceanogr* 2005;50:883–95. 10.4319/lo.2005.50.3.0883

[32] Long M, Marie D, Szymczak J. et al. Dinophyceae can use exudates as weapons against the parasite *Amoebophrya* sp. (Syndiniales). *ISME Com* 2021;1:34. 10.1038/s43705-021-00035-x PMC9723556 37938261

[33] Zheng B, Lucas AJ, Franks PJS. et al. Dinoflagellate vertical migration fuels an intense red tide. *Proc Natl Acad Sci U S A* 2023;120:e2304590120. 10.1073/pnas.230459012037639597 PMC10483641

[34] Cloern J, Dufford R. Phytoplankton community ecology: principles applied in San Francisco Bay. *Mar Ecol Prog Ser* 2005;285:11–28. 10.3354/meps285011

[35] Bulleri F . Facilitation research in marine systems: state of the art, emerging patterns and insights for future developments. *J Ecol* 2009;97:1121–30. 10.1111/j.1365-2745.2009.01567.x

[36] Picoche C, Barraquand F. Strong self-regulation and widespread facilitative interactions in phytoplankton communities. *J Ecol* 2020;108:2232–42. 10.1111/1365-2745.13410

[37] Blanchet FG, Cazelles K, Gravel D. Co-occurrence is not evidence of ecological interactions. *Ecol Lett* 2020;23:1050–63. 10.1111/ele.13525 32429003

[38] Landi P, Minoarivelo HO, Brännström Å. et al. Complexity and stability of ecological networks: a review of the theory. *Popul Ecol* 2018;60:319–45. 10.1007/s10144-018-0628-3

[39] Delmas E, Besson M, Brice M-H. et al. Analysing ecological networks of species interactions. *Biol Rev* 2019;94:16–36. 10.1111/brv.12433 29923657

[40] Trombetta T, Vidussi F, Roques C. et al. Co-occurrence networks reveal the central role of temperature in structuring the plankton community of the Thau lagoon. *Sci Rep* 2021;11:17675. 10.1038/s41598-021-97173-y34480057 PMC8417261

[41] Shen Y, Zhou X, Zhang J. et al. Insights into the effects of environmental factors on phytoplankton and microzooplankton at a basin scale: diversity, assembly mechanisms, and co-occurrence networks. *Front Mar Sci* 2024;11:1462432. 10.3389/fmars.2024.1462432

[42] Chaffron S, Delage E, Budinich M. et al. Environmental vulnerability of the global ocean epipelagic plankton community interactome. *Sci Adv* 2021;7:eabg1921. 10.1126/sciadv.abg192134452910 PMC8397264

[43] Deutschmann IM, Krabberød AK, Latorre F. et al. Disentangling temporal associations in marine microbial networks. *Microbiome* 2023;11:83. 10.1186/s40168-023-01523-z37081491 PMC10120119

[44] Costas-Selas C, Martínez-García S, Logares R. et al. Role of bacterial community composition as a driver of the small-sized phytoplankton community structure in a productive coastal system. *Microb Ecol* 2023;86:777–94. 10.1007/s00248-022-02125-236305941 PMC10335964

[45] REPHY - French Observation and Monitoring program for Phytoplankton and Hydrology in coastal waters . REPHY dataset - French observation and monitoring program for phytoplankton and hydrology in coastal waters. In: Metropolitan Data. SEANOE, 2023, 10.17882/47248.

[46] Belin C, Soudant D, Amzil Z. Three decades of data on phytoplankton and phycotoxins on the French coast: lessons from REPHY and REPHYTOX. *Harmful Algae* 2021;102:101733. 10.1016/j.hal.2019.101733 33875174

[47] Utermöhl H . Neue Wege in der quantitativen Erfassung des Planktons (mit besonderer Berücksichtigung des Ultraplanktons). *SIL Proc* 1931;5:567–96. 10.1080/03680770.1931.11898492

[48] Borja Á, Dauer DM, Elliott M. et al. Medium- and long-term recovery of estuarine and coastal ecosystems: patterns, rates and restoration effectiveness. *Estuar Coasts* 2010;33:1249–60. 10.1007/s12237-010-9347-5

[49] Lheureux A, David V, Del Amo Y. et al. Trajectories of nutrients concentrations and ratios in the French coastal ecosystems: 20 years of changes in relation with large-scale and local drivers. *Sci Total Environ* 2023;857:159619. 10.1016/j.scitotenv.2022.159619 36280086

[50] Josse J, Husson F. missMDA: a package for handling missing values in multivariate data analysis. *J Stat Softw* 2016;70:1–31. 10.18637/jss.v070.i01

[51] Chavent M, Kuentz-Simonet V, Labenne A. et al. ClustGeo: an R package for hierarchical clustering with spatial constraints. *Comput Stat* 2018;33:1799–822. 10.1007/s00180-018-0791-1

[52] Benjamini Y, Hochberg Y. On the adaptive control of the false discovery rate in multiple testing with independent statistics. *J Educ Behav Stat* 2000;25:60–83. 10.3102/10769986025001060

[53] Peschel S, Müller CL, von Mutius E. et al. NetCoMi: network construction and comparison for microbiome data in R. *Brief Bioinform* 2021;22:bbaa290. 10.1093/bib/bbaa29033264391 PMC8293835

[54] Shannon CE . A mathematical theory of communication. *Bell Syst Tech J* 1948;27:379–423. 10.1002/j.1538-7305.1948.tb01338.x

[55] Pielou EC . The measurement of diversity in different types of biological collections. *J Theor Biol* 1966;13:131–44. 10.1016/0022-5193(66)90013-0

[56] Berger WH, Parker FL. Diversity of planktonic foraminifera in deep-sea sediments. *Science* 1970;168:1345–7. 10.1126/science.168.3937.1345 17731043

[57] Cleveland RB, Cleveland WS, Terpenning I. STL: a seasonal-trend decomposition procedure based on loess. *J Off Stat* 1990;6:3.

[58] Leys C, Ley C, Klein O. et al. Detecting outliers: do not use standard deviation around the mean, use absolute deviation around the median. *J Exp Soc Psychol* 2013;49:764–6. 10.1016/j.jesp.2013.03.013

[59] R Core Team . R: A Language and Environment for Statistical Computing. Vienna, Austria: R Foundation for Statistical Computing, 2024, https://www.R-project.org/.

[60] Caracciolo M, Rigaut-Jalabert F, Romac S. et al. Seasonal dynamics of marine protist communities in tidally mixed coastal waters. *Mol Ecol* 2022;31:3761–83. 10.1111/mec.1653935593305 PMC9543310

[61] Caroppo C, Turicchia S, Margheri MC. Phytoplankton assemblages in coastal waters of the northern Ionian Sea (eastern Mediterranean), with special reference to cyanobacteria. *J Mar Biol Assoc UK* 2006;86:927–37. 10.1017/S0025315406013889

[62] Leblanc K, Arístegui J, Armand L. et al. A global diatom database – abundance, biovolume and biomass in the world ocean. *Earth Syst Sci Data* 2012;4:149–65. 10.5194/essd-4-149-2012

[63] Loreau M . Separating sampling and other effects in biodiversity experiments. *Oikos.* 1998;82:600. 10.2307/3546381

[64] Cermeño P, Teixeira IG, Branco M. et al. Sampling the limits of species richness in marine phytoplankton communities. *J Plankton Res* 2014;36:1135–9. 10.1093/plankt/fbu033

[65] Qian JJ, Akçay E. The balance of interaction types determines the assembly and stability of ecological communities. *Nat Ecol Evol* 2020;4:356–65. 10.1038/s41559-020-1121-x 32094535

[66] Lurgi M, Montoya D, Montoya JM. The effects of space and diversity of interaction types on the stability of complex ecological networks. *Theor Ecol* 2016;9:3–13. 10.1007/s12080-015-0264-x

[67] Slobodkin LB . The null case of the paradox of the plankton: after-dinner address at MSRC Conference on Blooms, October 27, 1988. In: Cosper E.M., Bricelj V.M., Carpenter E.J. (eds.), Coastal and Estuarine Studies, Vol. 35. Washington, D. C: American Geophysical Union, 1989, 10.1029/CE035p0341.

[68] Irigoien X, Flynn KJ, Harris RP. Phytoplankton blooms: a “loophole” in microzooplankton grazing impact? *J Plankton Res* 2005;27:313–21. 10.1093/plankt/fbi011

[69] Jeong HJ, Yoo YD, Seong KA. et al. Feeding by the mixotrophic red-tide dinoflagellate Gonyaulax polygramma: mechanisms, prey species, effects of prey concentration, and grazing impact. *Aquat Microb Ecol* 2005;38:249–57. 10.3354/ame038249

[70] Park MG, Kim S, Kim HS. et al. First successful culture of the marine dinoflagellate Dinophysis acuminata. *Aquat Microb Ecol* 2006;45:101–6. 10.3354/ame045101

[71] Daro MH, Breton E, Antajan E. et al. Do Phaeocystis colony blooms affect zooplankton in the Belgian coastal zone? In: Rousseau V., Lancelot C., Cox D. (eds.), Current Status of Eutrophication in the Belgian Coastal Zone. Bruxelles: Presses Universitaires de Bruxelles, 2008, 61–72.

[72] Gasparini S, Daro MH, Antajan E. et al. Mesozooplankton grazing during the Phaeocystis globosa bloom in the southern Bight of the North Sea. *J Sea Res* 2000;43:345–56. 10.1016/S1385-1101(00)00016-2

[73] Zhang H, Wang K, Shen L. et al. Microbial community dynamics and assembly follow trajectories of an early-spring diatom bloom in a semienclosed bay. *Appl Environ Microbiol* 2018;84:e01000–18. 10.1128/AEM.01000-1830006403 PMC6121991

[74] Hou F, Zhang H, Xie W. et al. Co-occurrence patterns and assembly processes of microeukaryotic communities in an early-spring diatom bloom. *Sci Total Environ* 2020;711:134624. 10.1016/j.scitotenv.2019.134624 31818596

[75] Wyatt T . Margalef’s mandala and phytoplankton bloom strategies. *Deep Sea Res Part II Top Stud Oceanogr* 2014;101:32–49. 10.1016/j.dsr2.2012.12.006

[76] Mitra A, Flynn KJ. Predator–prey interactions: is “ecological stoichiometry” sufficient when good food goes bad? *J Plankton Res* 2005;27:393–9. 10.1093/plankt/fbi022

[77] Poulin FJ, Franks PJS. Size-structured planktonic ecosystems: constraints, controls and assembly instructions. *J Plankton Res* 2010;32:1121–30. 10.1093/plankt/fbp14520625560 PMC2900175

[78] Hamm C, Smetacek V. Armor: Why, when, and how. In: Falkowski P.G., Knoll A.H. (eds.), Evolution of Primary Producers in the Sea. Elsevier, 2007, 311–32 10.1016/B978-012370518-1/50015-1.

[79] Odum E, Barett G. Fundamentals of Ecology, 3rd edn. Philadelphia: Saunders, 1971.

[80] Loehle C . Challenges of ecological complexity. *Ecol Complex* 2004;1:3–6. 10.1016/j.ecocom.2003.09.001

[81] Jochimsen MC, Kümmerlin R, Straile D. Compensatory dynamics and the stability of phytoplankton biomass during four decades of eutrophication and oligotrophication. *Ecol Lett* 2013;16:81–9. 10.1111/ele.12018 23050937

[82] Gonzalez A, Loreau M. The causes and consequences of compensatory dynamics in ecological communities. *Annu Rev Ecol Evol Syst* 2009;40:393–414. 10.1146/annurev.ecolsys.39.110707.173349

[83] Biggs CR, Yeager LA, Bolser DG. et al. Does functional redundancy affect ecological stability and resilience? A review and meta-analysis. *Ecosphere* 2020;11:e03184. 10.1002/ecs2.3184

[84] Pan Q, Tian D, Naeem S. et al. Effects of functional diversity loss on ecosystem functions are influenced by compensation. *Ecology* 2016;97:2293–302. 10.1002/ecy.1460 27859077

[85] Alexander TJ, Vonlanthen P, Seehausen O. Does eutrophication-driven evolution change aquatic ecosystems? *Philos Trans R Soc B Biol Sci* 2017;372:20160041. 10.1098/rstb.2016.0041 PMC5182437 27920386

[86] Cupertino A, Gücker B, Von Rückert G. et al. Phytoplankton assemblage composition as an environmental indicator in routine lentic monitoring: taxonomic versus functional groups. *Ecol Indic* 2019;101:522–32. 10.1016/j.ecolind.2019.01.054

[87] Salmaso N, Naselli-Flores L, Padisák J. Functional classifications and their application in phytoplankton ecology. *Freshw Biol* 2015;60:603–19. 10.1111/fwb.12520

[88] Hanski I . Dynamics of regional distribution: the core and satellite species hypothesis. *Oikos.* 1982;38:210. 10.2307/3544021

[89] Jenkins MF, White EP, Hurlbert AH. The proportion of core species in a community varies with spatial scale and environmental heterogeneity. *PeerJ.* 2018;6:e6019. 10.7717/peerj.601930533308 PMC6276595

[90] Gibson DJ, Ely JS, Collins SL. The core–satellite species hypothesis provides a theoretical basis for Grime’s classification of dominant, subordinate, and transient species. *J Ecol* 1999;87:1064–7. 10.1046/j.1365-2745.1999.00424.x

[91] Xue Y, Chen H, Xiao P. et al. Core taxa drive microeukaryotic community stability of a deep subtropical reservoir after complete mixing. *Environ Microbiol Rep* 2023;15:769–82. 10.1111/1758-2229.1319637688478 PMC10667671

[92] Helden AJ . Core and occasional species: a new way forward. *Ecol Evol* 2021;11:10547–65. 10.1002/ece3.786334367596 PMC8328456

[93] Custer GF, Gans M, Van Diepen LTA. et al. Comparative analysis of core microbiome assignments: implications for ecological synthesis. *mSystems.* 2023;8:e01066–22. 10.1128/msystems.01066-2236744955 PMC9948721

[94] Deutschmann IM, Delage E, Giner CR. et al. Disentangling microbial networks across pelagic zones in the tropical and subtropical global ocean. *Nat Commun* 2024;15:126. 10.1038/s41467-023-44550-y38168083 PMC10762198

[95] Terry JCD, Bonsall MB, Morris RJ. The impact of structured higher-order interactions on ecological network stability. *Theor Ecol* 2025;18:9. 10.1007/s12080-025-00603-041541601 PMC12799688

[96] Jackson Z, Xue B. Heterogeneity of interaction strengths and its consequences on ecological systems. *Sci Rep* 2023;13:1905. 10.1038/s41598-023-28473-836732566 PMC9895049

[97] Gross T, Rudolf L, Levin SA. et al. Generalized models reveal stabilizing factors in food webs. *Science* 2009;325:747–50. 10.1126/science.1173536 19661430

[98] Gleich SJ, Mesrop LY, Cram JA. et al. With a little help from my friends: importance of protist-protist interactions in structuring marine protistan communities in the San Pedro Channel. *mSystems* 2025;10:e0104524–4. 10.1128/msystems.01045-2439878540 PMC11834403

[99] Dunne JA, Williams RJ, Martinez ND. Network structure and biodiversity loss in food webs: robustness increases with connectance. *Ecol Lett* 2002;5:558–67. 10.1046/j.1461-0248.2002.00354.x

[100] Dunne JA, Williams RJ. Cascading extinctions and community collapse in model food webs. *Philos Trans R Soc B* 2009;364:1711–23. 10.1098/rstb.2008.0219 PMC2685420 19451122

[101] Allesina S, Tang S. Stability criteria for complex ecosystems. *Nature* 2012;483:205–8. 10.1038/nature10832 22343894

[102] Carpentier C, Barabás G, Spaak JW. et al. Reinterpreting the relationship between number of species and number of links connects community structure and stability. *Nat Ecol Evol* 2021;5:1102–9. 10.1038/s41559-021-01468-2 34059819

[103] Thébault E, Fontaine C. Stability of ecological communities and the architecture of mutualistic and trophic networks. *Science* 2010;329:853–6. 10.1126/science.1188321 20705861

[104] Oldenburg EM, Kronberg RM, Metfies K. et al. Beyond blooms: the winter ecosystem reset determines microeukaryotic community dynamics in the Fram Strait. *Commun Earth Environ* 2024;5:643. 10.1038/s43247-024-01782-0

