# Supplementary material for: Diatoms vs dinoflagellates: a temporal network analysis of bloom impacts on phytoplankton diversity and community structure in French coastal waters
Source: ISME Commun. 2026 Jun 19;6(1):ycag174. doi: 10.1093/ismeco/ycag174 (PMC13374866; doi:10.1093/ismeco/ycag174)
Supplement: Supplementary_M_Diatoms_vs_dinoflagellates_vRview_final_ycag174 [file supplementary_m_diatoms_vs_dinoflagellates_vrview_final_ycag174.docx]

**Supplementary materials**

**Diatoms vs dinoflagellates: a temporal network analysis of bloom impacts on phytoplankton diversity and community structure in French coastal waters**

Jean-Yves Dias^a,b^, Victor Pochic^a,c^, Samuel Chaffron^d,e^, Pierre Gernez^a^

^a^Nantes Université, Institut des Substances et Organismes de la Mer, ISOMER, UR 2160, F-44000 Nantes, France ^b^Laboratoire de Biologie des Organismes et des Écosystèmes Aquatiques-BOREA, Muséum national d’Histoire naturelle (MNHN), SU, CNRS, IRD, UA, F-75005 Paris, France ^c^Ifremer, COAST, F-44000 Nantes, France ^d^Nantes Université, École Centrale Nantes, CNRS, LS2N, UMR 6004, F-44000 Nantes, France ^e^Research Federation for the Study of Global Ocean Systems Ecology and Evolution, FR2022/Tara Oceans GOSEE, F-75016 Paris, France

Corresponding authors: diasjeanyves@yahoo.com (J-Y. Dias), samuel.chaffron@cnrs.fr (S. Chaffron), pierre.gernez@univ-nantes.fr (P. Gernez)


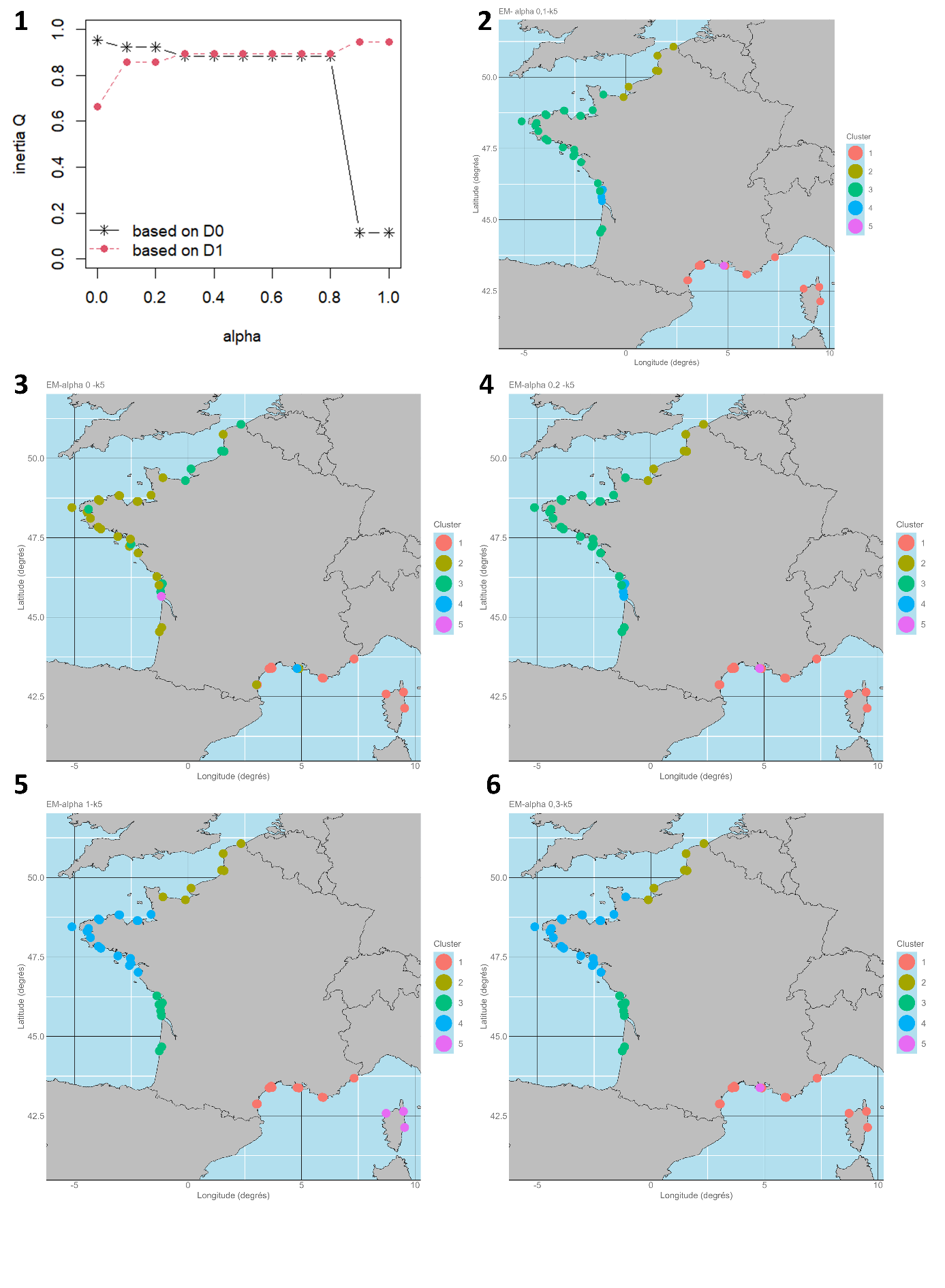


**Supp. Mat. 1 : More details on the choice of the weight for spatial constraints used for regionalization.** Panel 1 shows the proportion of explained pseudo-inertia (Q, y-axis) calculated with hydrological distances (D0, black solid line) and with geographical distances (D1, dashed line) for different alpha values (x-axis). Panel 2 shows the regionalization based on 10% (as chosen in the manuscript), and panels 3, 4, 5 and 6 respectively show the regionalization based on pure hydrology (0%), 20%, 30% spatial constraints, and pure spatial coherence (100%).

Our stations cover all mainland French coastlines, which differ in biological and physicochemical ways. To study the broader geographical area beyond each individual station, a hydrology-based regionalization that considers the proximities between them was conducted. The workflow to do it is presented in the Materials & Methods “Hydrology-based regionalization” section and corresponds to the one developed by Chavent et al. (2018) using the ClustGeo R package. The key parameter for this analysis is the choice of the weight for spatial constraint; it gives more or less weight to the geographical clustering than to the hydrology clustering. The objective of this Supplementary Material is to show how this parameter was set to 10%.

Chavent et al. (2018) recommend selecting this parameter (named alpha) as a trade-off between [hydrology] proximity and spatial cohesion. To help in choosing it, the function choicealpha() plots the proportion of explained pseudo-inertia (Q, y-axis) calculated with hydrological distances (D0, black solid line) and with geographical distances (D1, dashed line) for different alpha values (x-axis). This figure is presented in panel 1. For our data, it appears clearly that selecting a weight of 10% (alpha = 0.1) is the best trade-off to improve spatial cohesion without affecting hydrological cohesion.

We made some sensitivity analyses to ensure this. Panel 2 shows the different clusters (i.e. regions) that we obtained and used in the study. Using 46 sampling stations for which the length of the Chl-a time series without a 2-month gap was longer than 5 years (except during the COVID-19 pandemic), we obtained 6 clusters. The first cluster (red), second (khaki) and third (green) correspond respectively to the Mediterranean Sea, Atlantic Ocean-Western Channel and Eastern Channel. The fourth cluster corresponds to stations belonging to the Pertuis Sea and was not used in our study because of an abrupt change in species richness that we inferred to be due to a change in sampling analysis design. The fifth region corresponds to the “Grand Rhône” station, which is very close to the mouth of the Rhône River and therefore has very low salinity, which explains its uniqueness. Panel 3 shows the hydrology regionalization (i.e. alpha = 0). This clustering shows the limits of using only hydrological information for regionalization, as it clusters one Mediterranean station within the Atlantic cluster and some very distant Atlantic stations within the Eastern Channel cluster. As the objective is also to have a relatively coherent community, it is clear that the Mediterranean phytoplankton community is totally different from the Atlantic one. It also appears that the stations in the Atlantic which belong to the Eastern Channel are more influenced by the same environments (e.g. tide) and pressures than those belonging to the Atlantic one. Panel 4 shows the regionalization based on alpha 0.2; the results are identical to the chosen 0.1. Panel 5 shows the regionalization based on a weight of 30% for spatial constraint. The main difference is that the southernmost Atlantic stations belong to the same cluster as those of the Pertuis Sea. The Pertuis Sea is known to be a semi-closed area with a high fluvial input and not very sensitive to tides. On the contrary, the southernmost stations have an oceanic influence. Panel 6 shows the geographical regionalization (alpha = 1). As expected, it is very “geographic”, as Corsica differs from the continental Mediterranean stations, the Grand Rhône station is no longer distinct, and we find again the Pertuis Sea region with the southernmost Atlantic stations. These results highlight that we lost some hydrological differences since a weight of 0.3 for geographical constraints is chosen; this is also coherent with the lower explained pseudo-inertia based on hydrology.

Altogether, this means that our choice of 10% for geographical constraints allows us to blur some little hydrological differences for very close stations, and we can hypothesize that they are more similar if we take into account parameters that are not in the data. Similarly, stations that share very close hydrological environments are more distinct if we take into account their different local geographical environments when they are far from each other. This is also supported by the large geographical area that we study. One question that can arise is why computing all these analyses to distinguish the Mediterranean Sea, Atlantic and English Channel. The answer appears in the limit between the Atlantic and the English Channel that delimits the Western and Eastern parts of the Channel. It is also a more objective regionalization based on data. Moreover, the limit between the Western and Eastern Channel is also found by Beaugrand et al. (2019) [10.1016/j.pocean.2019.02.014] using an ecological partition of the North Atlantic Ocean and the CPR survey data.

Note that there are more stations in this Supplementary Material than in the manuscript because the regionalization is based on 46 sampling stations for which the length of the chla time series without a 2-month gap was longer than 5 years (except during the COVID-19 pandemic), whereas the study is based on a second selection retaining, from these 46 stations, 21 chla time series with at least 15 years of data within the same period, i.e., from March 2007 to August 2022.

| A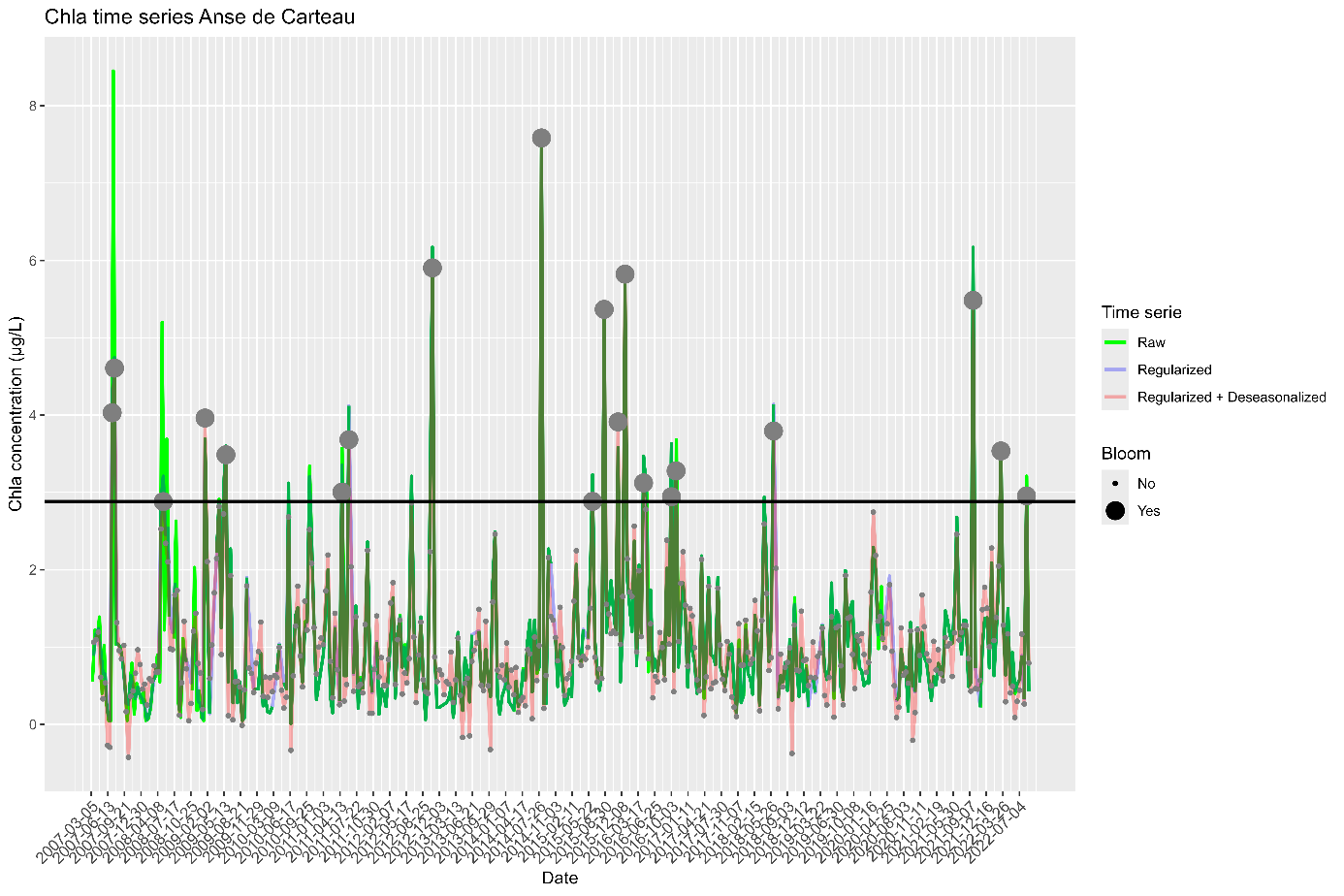 |
| --- |
| B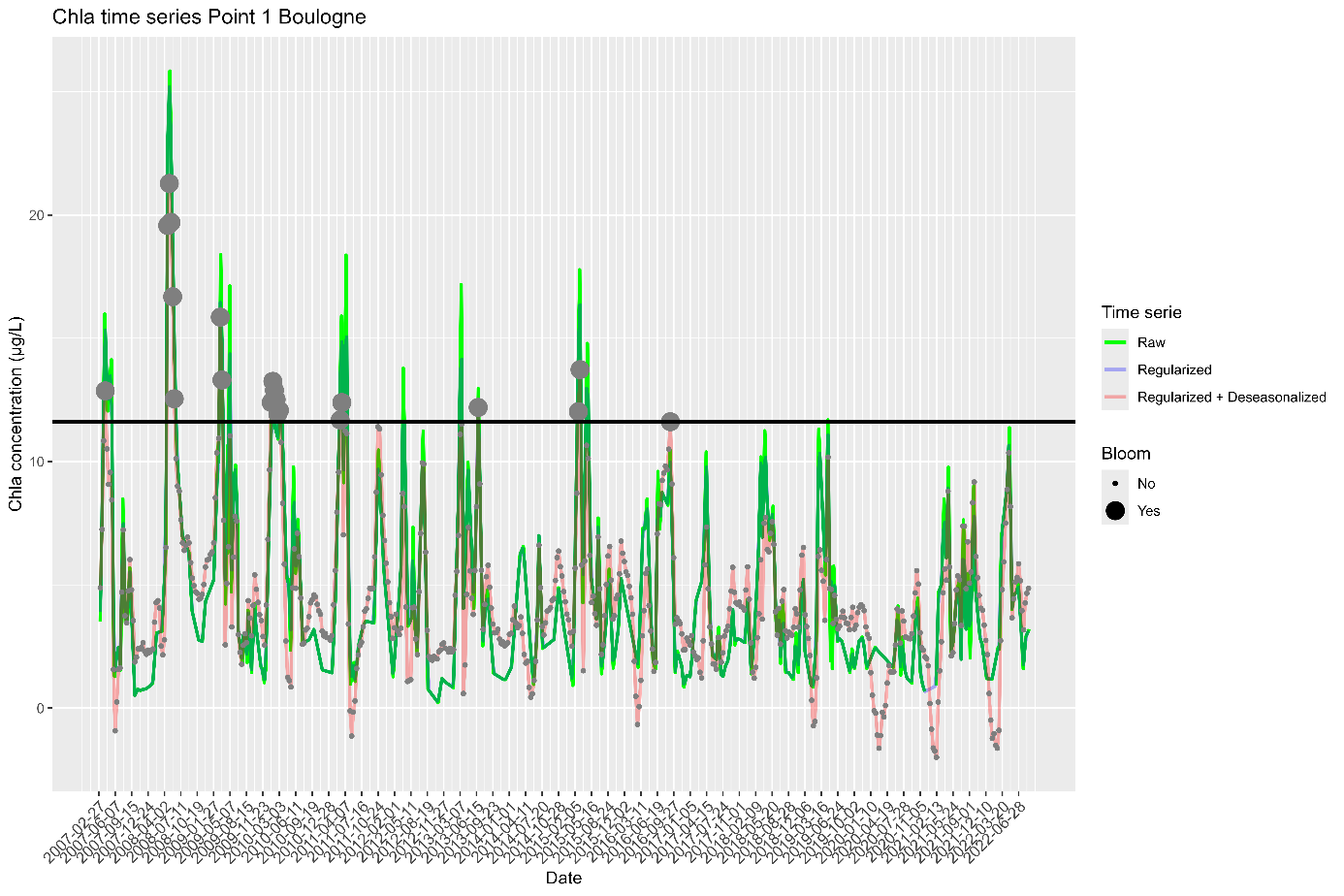 |
| C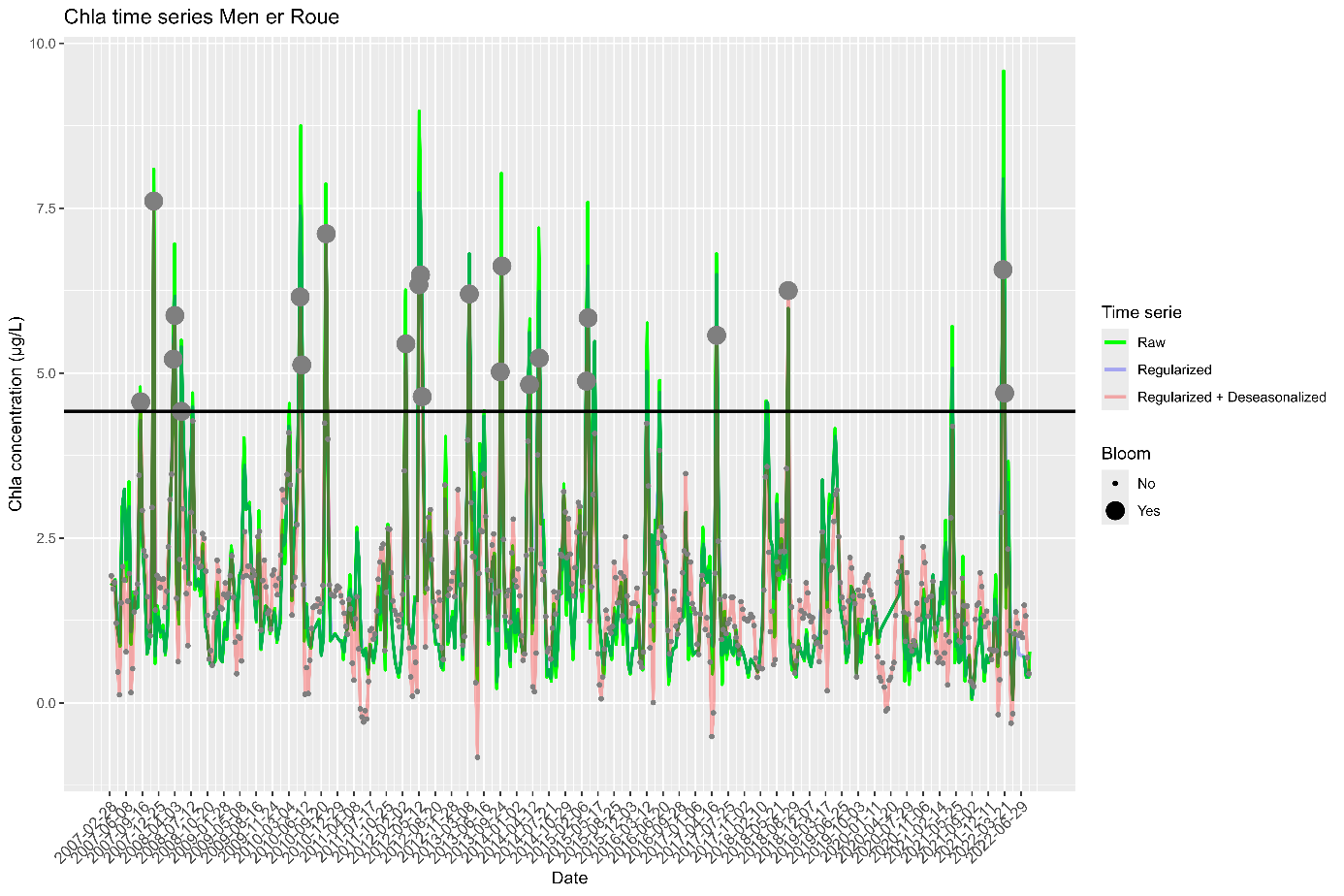 |

**Supp. Mat. 2 : More details into the bloom detection method.**

In some definitions, outliers are points whose values are 2 or 3 times greater than the standard deviation around the mean, although the mean and standard deviation are themselves sensitive to outliers. An alternative is to use the absolute deviation from the median (MAD, Leys et al., 2013) in order to limit the influence of outliers. A relationship adapted from the work of Leys et al. (2013) was used: MAD = 1.486a × M(|x − M(x)|), where x corresponds to the values of the chla time series and M to the median. The value 1.486 is defined so that the MAD is equivalent to the standard deviation for a normal distribution. We set a to 2.32 to match this value to the 99th quantile of a normal distribution, approximating the concept of a p-value. Blooms correspond to episodes where chla is extremely high compared to the baseline level of the series, satisfying the condition: |x − M(x)| > 2.32 x 1.486 × MAD.

This method is more objective, as the threshold used to detect blooms is not chosen manually by looking at the time series because it “works,” as is often done, but is instead auto-adaptive to the distribution of each time series, using the same criterion for all, which is important given the different ranges of chla across regions. Indeed, there are many other ways to detect outliers, but our goal here is to study them, not to discard them, so this statistical method is the least influenced by outliers while remaining based on distributions. The figures display the time series in their raw, regularized, and regularized-and-deseasonalized forms, as well as the identified blooms. The horizontal line indicates the threshold above which values are considered outliers/blooms.


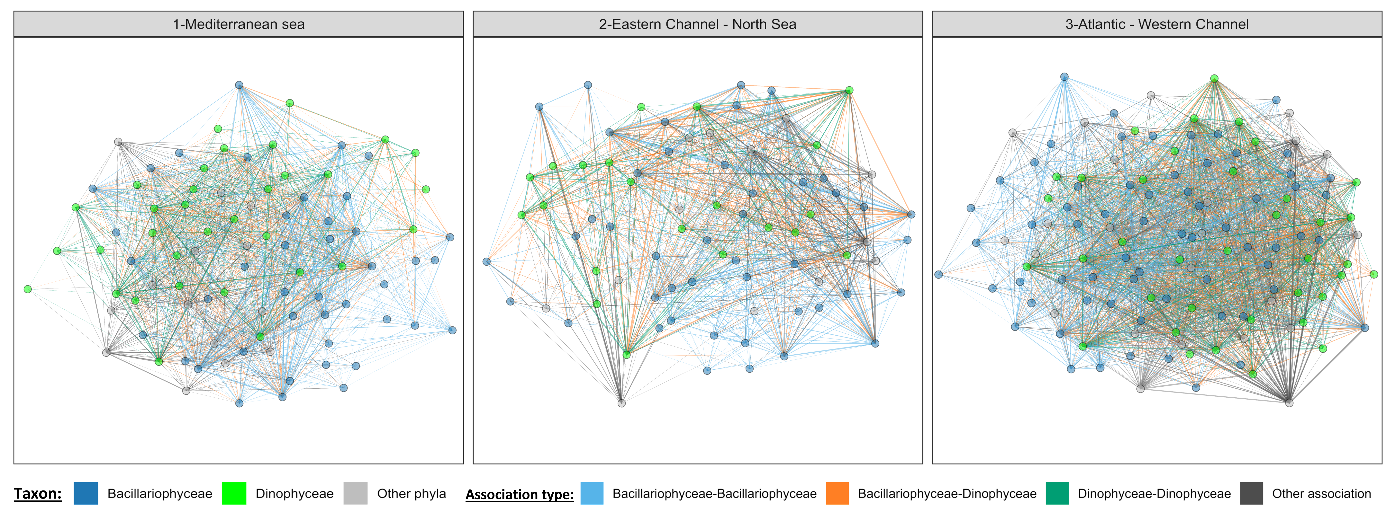


**Supp. Fig. 1 :** **Global association networks by region.** Nodes represent *Bacillariophyceae* (dark blue), *Dinophyceae* (green), or other phyla (grey). Links are coloured according to the type of association: associations between *Bacillariophyceae* (light blue), between *Dinophyceae* (dark green), between *Bacillariophyceae* and *Dinophyceae* (orange), or other types of associations (dark). Thicker links indicate stronger associations.


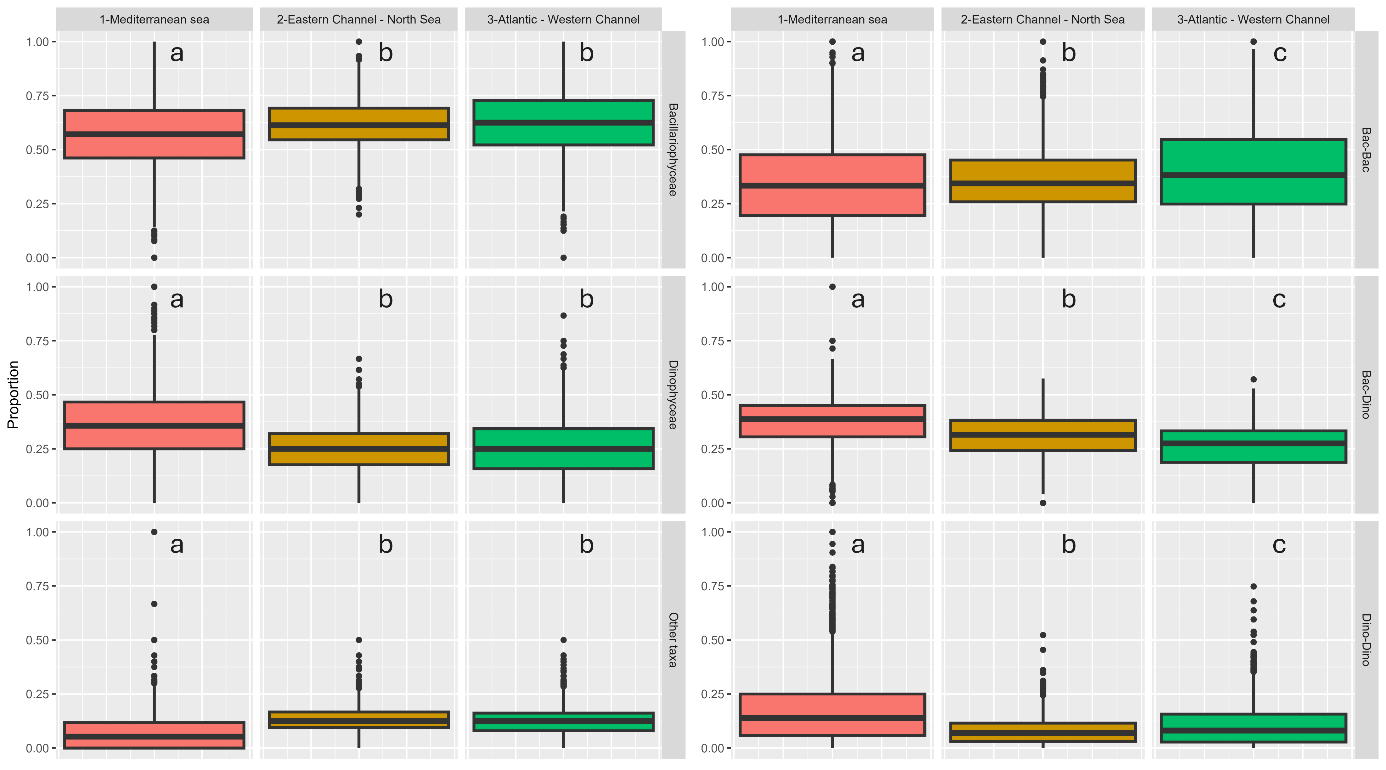


**Supp. Fig. 2: Differences between regions for the taxonomic (A) and association types (B) composition of the temporal association networks.** Letters indicate significant differences (p < 0.05) according to a Kruskal-Wallis test.


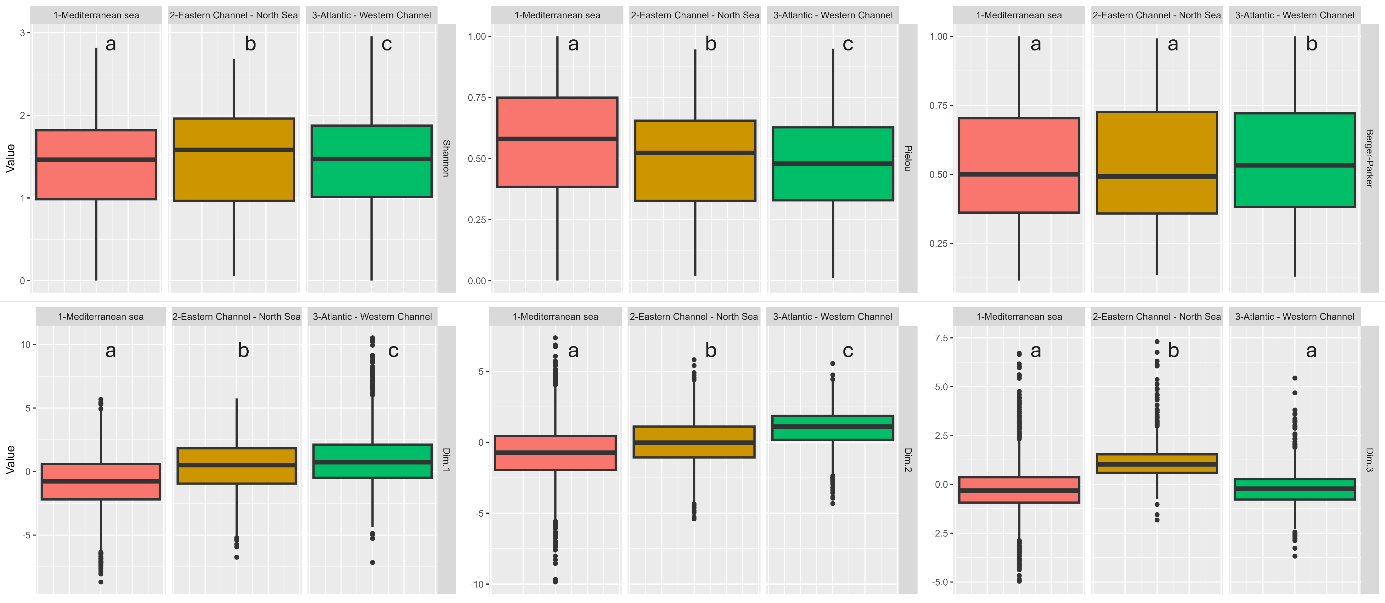


**Supp. Fig. 3:** **Differences between regions for diversity indices (upper part) and PCA dimensions based on graph metrics (lower part).** Letters indicate significant differences (p < 0.05) according to a Kruskal-Wallis test.


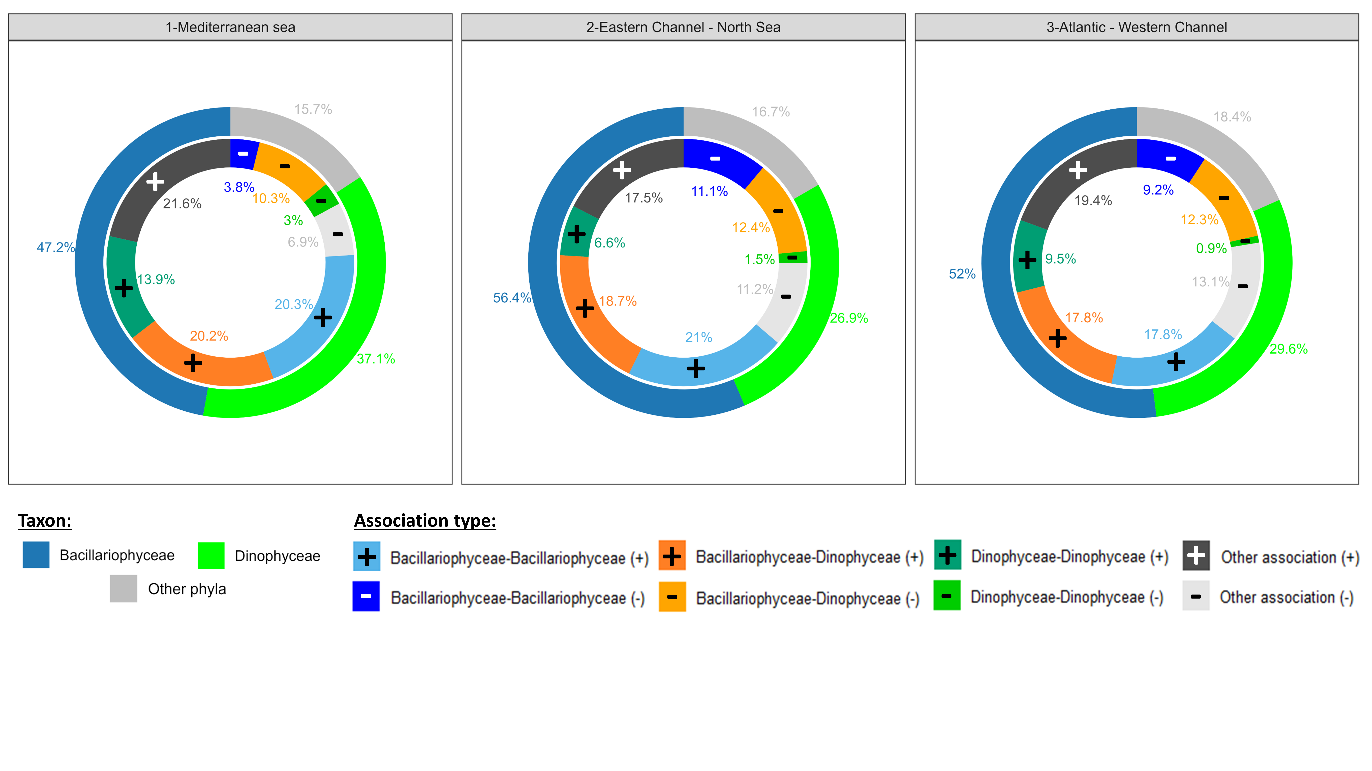


**Supp. Fig. 4:** **Global association networks composition by region.**  The outer circle represents the relative number of each taxon in the global network, *Bacillariophyceae* (dark blue), *Dinophyceae* (green), or other phyla (grey). The inner circle indicates the relative proportion of each association type, including negative associations. Positive and negative associations between *Bacillariophyceae* (blue), between *Dinophyceae* (green), between *Bacillariophyceae* and *Dinophyceae* (orange), or other types of associations (dark/grey).


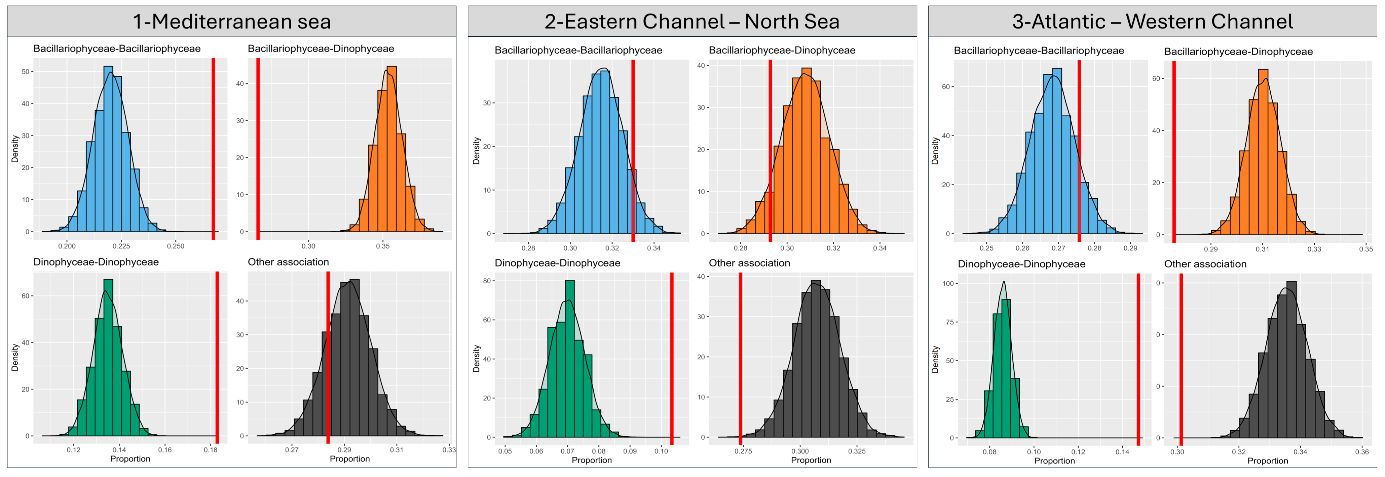
 **Supp. Fig. 5: Distribution of each association type proportion obtained with the random association networks (bar plots) and the observed association type proportion (red vertical line) in the global networks for each region.** The bar plots are colored according to the association type (between *Bacillariophyceae*, light blue; between *Bacillariophyceae* and *Dinophyceae*, orange; between *Dinophyceae*, dark green; and other types of associations, dark).


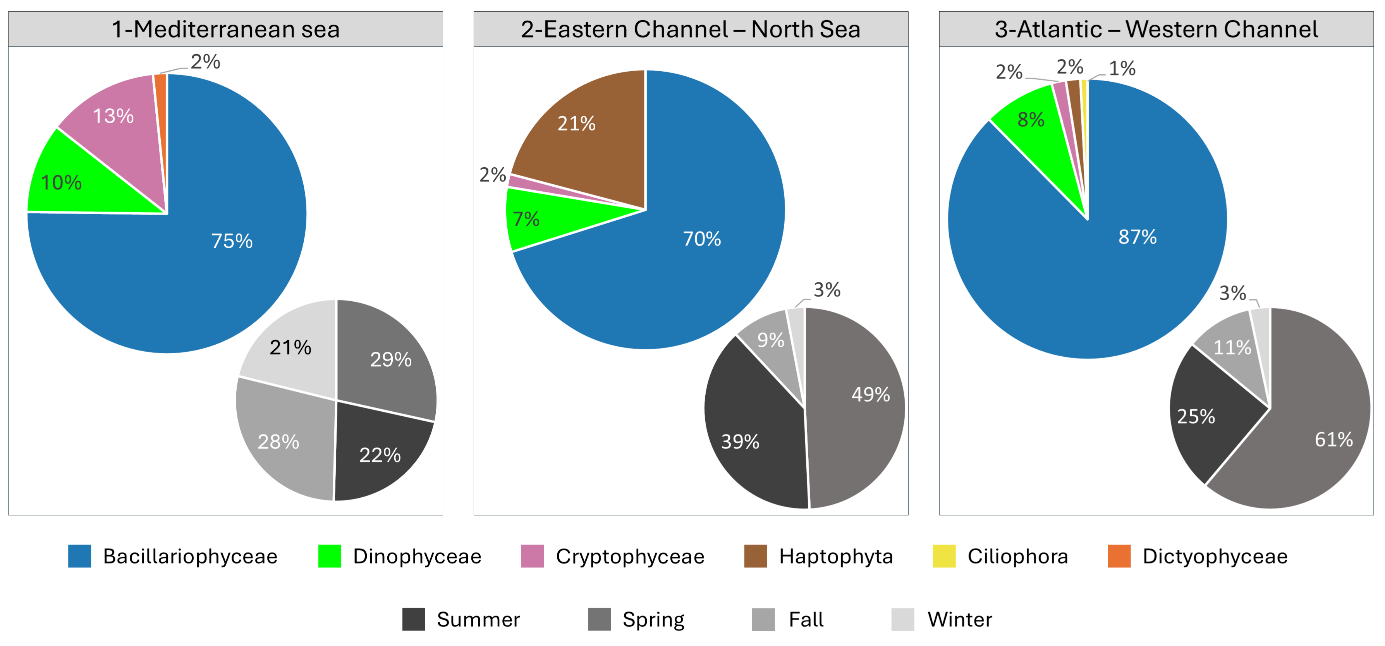
 **Supp. Fig. 6:** **Composition of the detected blooms by region.** The large colored pie chart shows the relative proportion of blooms detected by class, while the smaller grayscale pie chart displays the relative proportion of blooms detected by season.


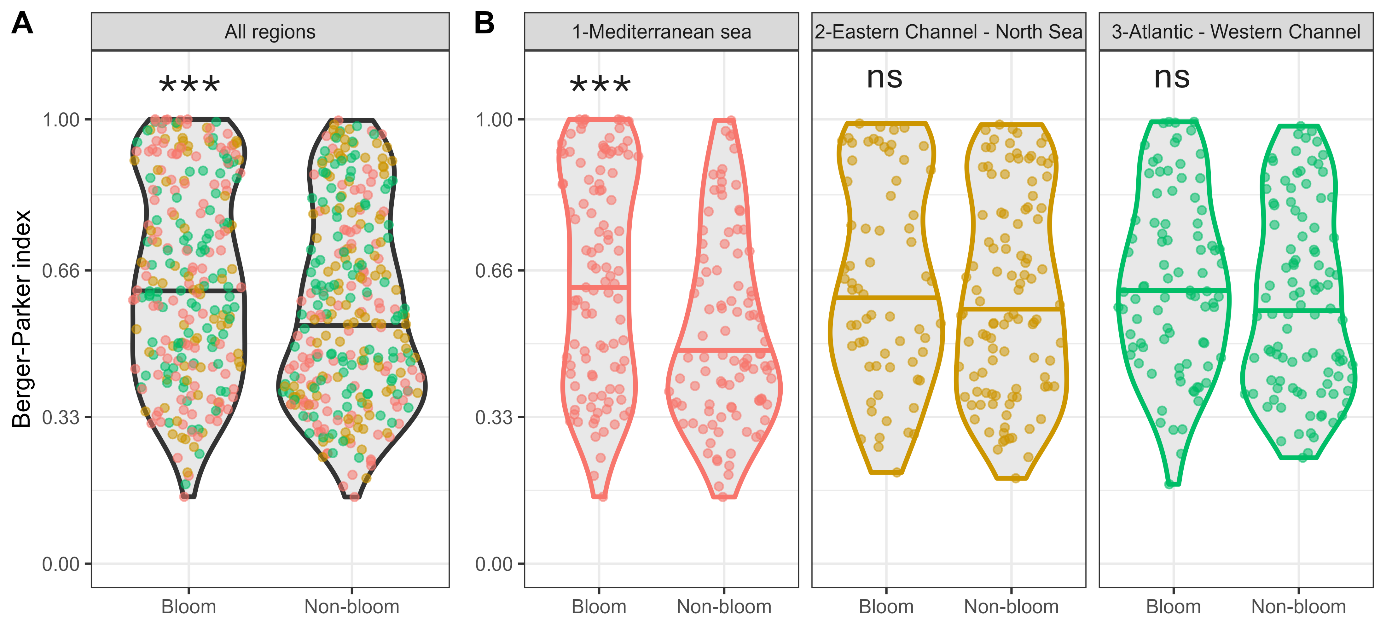
 **Supp. Fig. 7: Berger-Parker index during bloom and “non-bloom” sampling for all regions (A) and by region (B).** The horizontal line represents the mean value. Points and violin plots are colored according to the region (Mediterranean Sea, red; Eastern Channel–North Sea, brown; Atlantic–Western Channel, green). Asterisks indicate whether the Berger-Parker index during blooms is significantly different from “non-bloom” periods according to a Wilcoxon test (***: p < 0.0001, **: p < 0.001, *: p < 0.05, ns: p > 0.05).


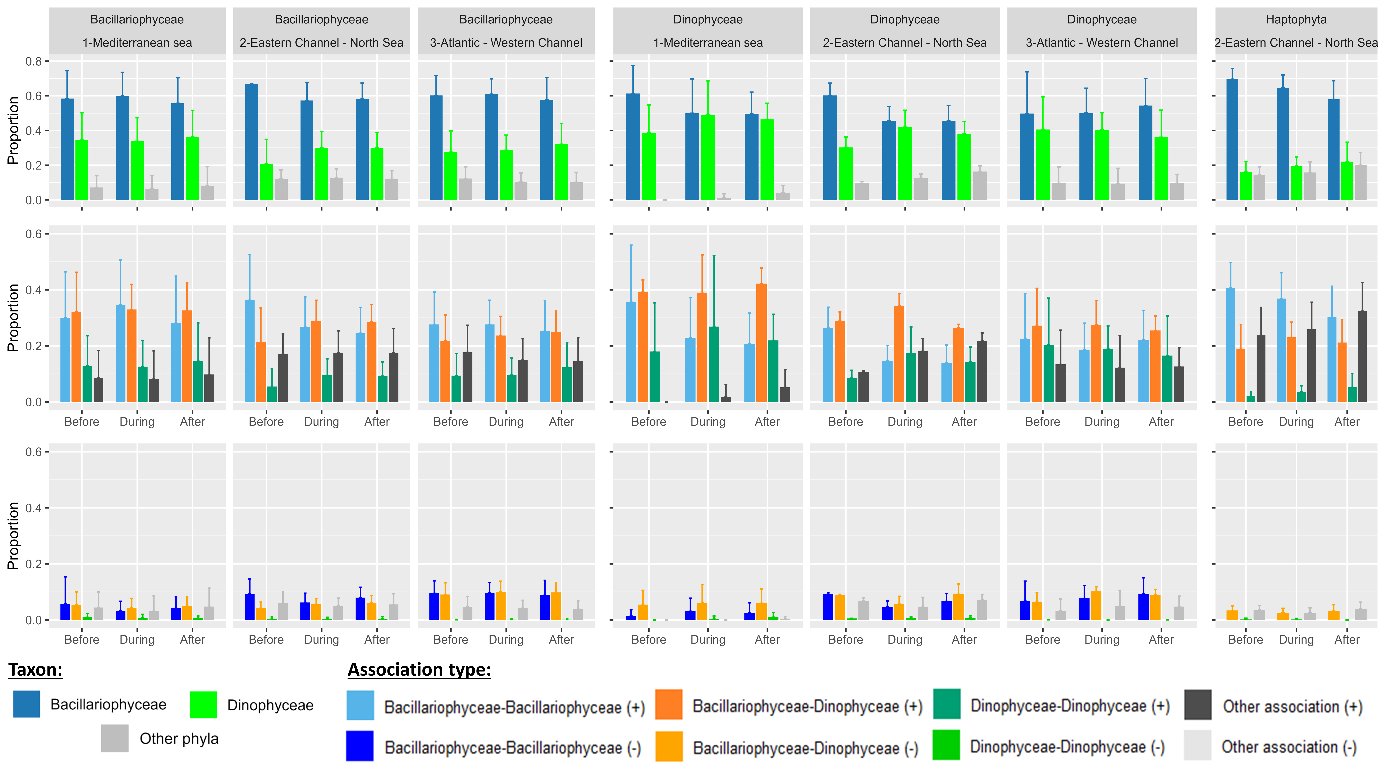
**Supp. Fig. 8:** **Composition of negative association networks before, during, and after Bacillariophyceae, Dinophyceae, and Haptophyta blooms in each region, expressed as a proportion of all associations (positive and negative).** The top bar plots show the average proportion of each taxon on the association networks (*Bacillariophyceae*, dark blue; *Dinophyceae*, green; other phyla, grey). The bottom bar plots display the average proportion of each association type (between *Bacillariophyceae*, light blue; between *Bacillariophyceae* and *Dinophyceae*, orange; between *Dinophyceae*, dark green; and other types of associations, dark). Error bars represent the standard deviation.
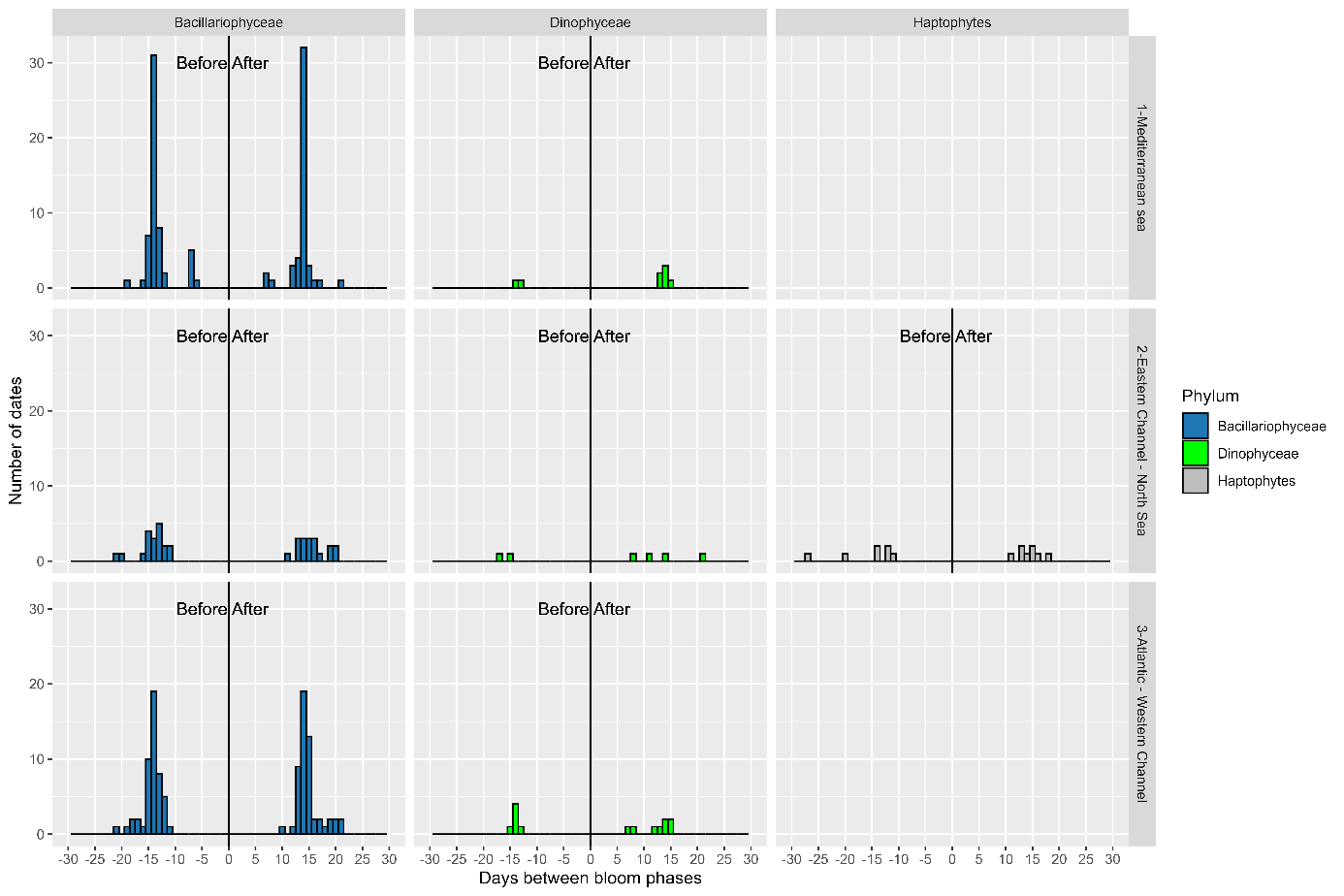
**Supp Fig.9 : Distribution of the time span between bloom phases for each region and bloom type.**

**Supp Table 1. Number of sampling dates for each region and bloom type, across bloom phases.**

| **1- Mediterranean Sea** | | | | |
| --- | --- | --- | --- | --- |
|  | Before | During | After | Total |
| Diatoms | 59 | 77 | 55 | 191 |
| Dinoflagellates | 5 | 13 | 6 | 24 |
| Haptophytes | 0 | 0 | 0 | 0 |
| Total | 64 | 90 | 61 | 215 |
| **2-Eastern Channel – North Sea** | | | | |
|  | Before | During | After | Total |
| Diatoms | 29 | 47 | 28 | 104 |
| Dinoflagellates | 2 | 5 | 4 | 11 |
| Haptophytes | 9 | 14 | 8 | 31 |
| Total | 40 | 66 | 40 | 146 |
| **3-Atlantic – Western Channel** | | | | |
|  | Before | During | After | Total |
| Diatoms | 55 | 74 | 55 | 184 |
| Dinoflagellates | 7 | 10 | 8 | 25 |
| Haptophytes | 0 | 0 | 0 | 0 |
| Total | 62 | 84 | 63 | 209 |
